# Supplementary figures and images for: Defining Reference Sequences for Nocardia Species by Similarity and Clustering Analyses of 16S rRNA Gene Sequence Data
Source: PLoS One. 2011 Jun 8;6(6):e19517. doi: 10.1371/journal.pone.0019517 (PMC3110597; doi:10.1371/journal.pone.0019517)

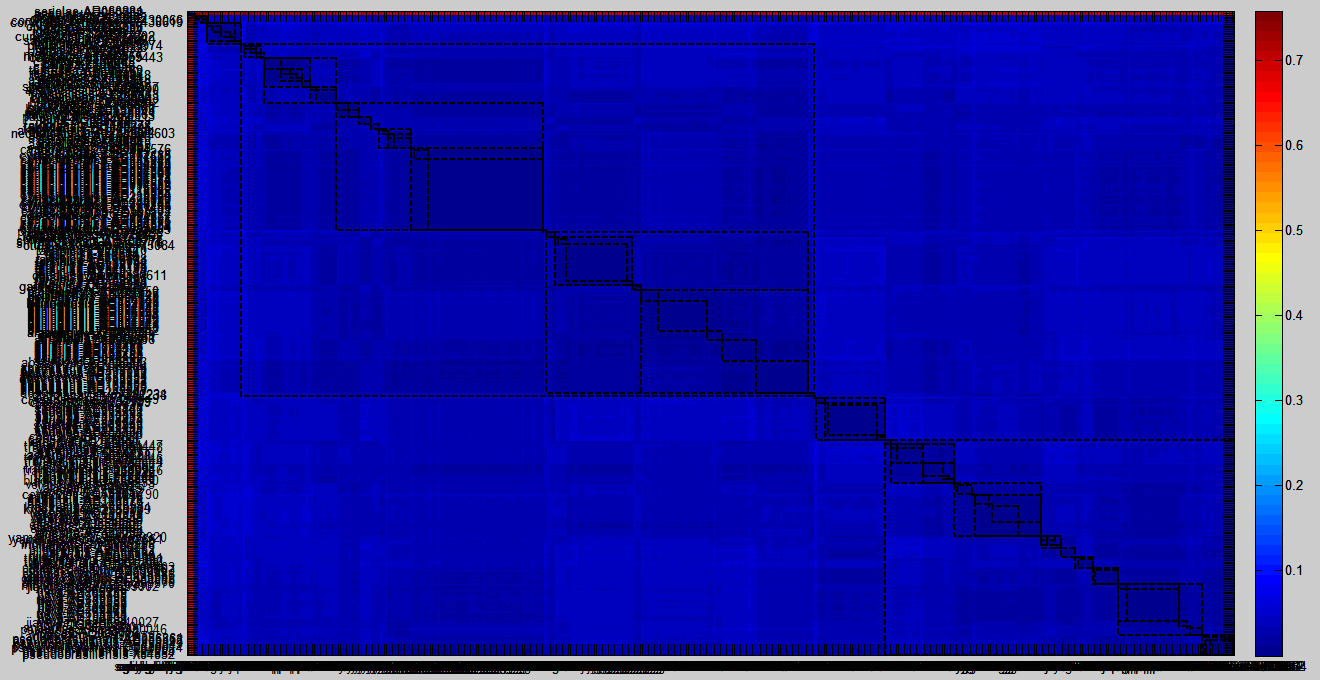

Supplement: Figure S1 — Heat map of the Nocardia distance matrix based on the Muscle alignment, showing rectangles around potential clusters identified by the Linear Mapping with different sensitivity parameters. (TIF) [file pone.0019517.s001.tif]

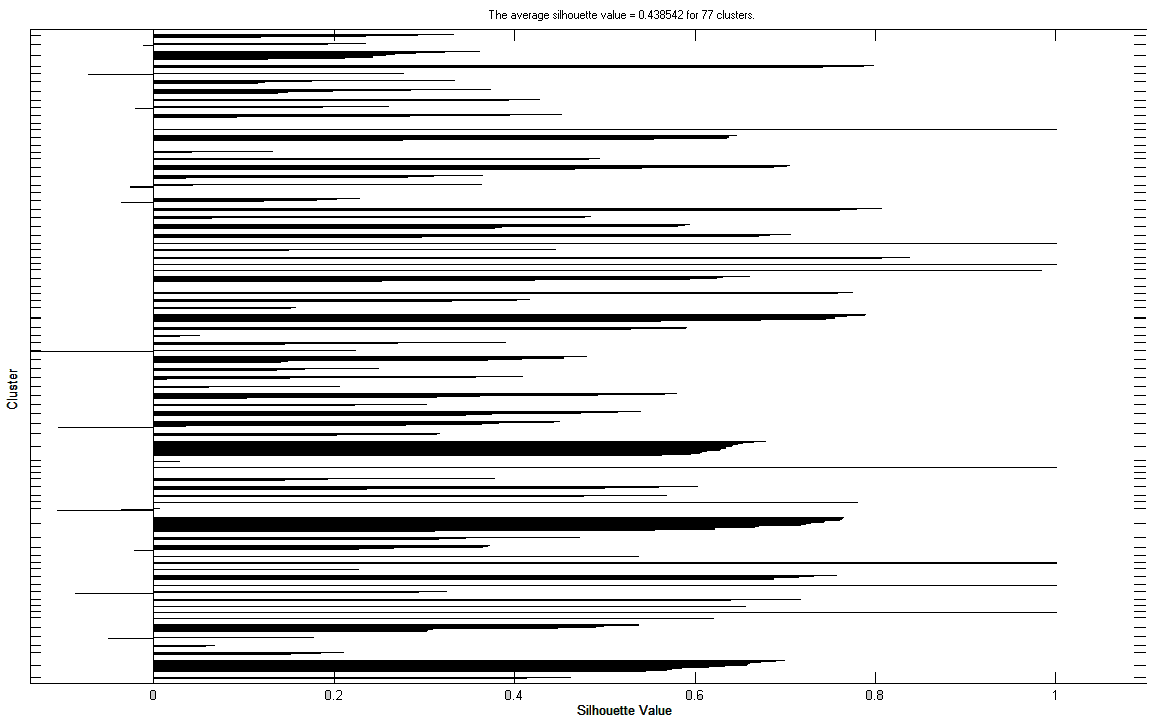

Supplement: Figure S2 — Silhouette values for 77 clusters defined by k-means clustering. (TIF) [file pone.0019517.s002.tif]
