# Supplementary material for: Defining Reference Sequences for Nocardia Species by Similarity and Clustering Analyses of 16S rRNA Gene Sequence Data
Source: PLoS One. 2011 Jun 8;6(6):e19517. doi: 10.1371/journal.pone.0019517 (PMC3110597; doi:10.1371/journal.pone.0019517)
Supplement: Table S1 — Total numbers of clusters generated by the linear mapping algorithms using different parameters. (DOC) [file pone.0019517.s003.doc]

**Table S1.** Number of total clusters generated based on the linear mapping algorithms using different parameters

| Hash Ranges | One Index /Cluster | Two Indices /Cluster | Three Indices /Cluster | Four Indices /Cluster |
| --- | --- | --- | --- | --- |
| 64 | 51 | 33 | 16 | 8 |
| 128 | 80 | 47 | 37 | 33 |
| 256 | 108 | 79 | 59 | 47 |
| 512 | 124 | 106 | 91 | 77 |
| 1024 | 142 | 121 | 91 | 77 |
| 2048 | 160 | 141 | 129 | 120 |
| 4096 | 160 | 160 | 159 | 141 |
| 8192 | 160 | 160 | 160 | 160 |
| 16384 | 160 | 160 | 160 | 160 |
| 32768 | 160 | 160 | 160 | 160 |
| 65536 | 160 | 160 | 160 | 160 |
| 131072 | 160 | 160 | 160 | 160 |
