# Supplementary material for: Defining Reference Sequences for Nocardia Species by Similarity and Clustering Analyses of 16S rRNA Gene Sequence Data
Source: PLoS One. 2011 Jun 8;6(6):e19517. doi: 10.1371/journal.pone.0019517 (PMC3110597; doi:10.1371/journal.pone.0019517)
Supplement: Table S2 — Linear mapping clustering using different colour map sizes and up to 4 colours per cluster for each map size. (DOC) [file pone.0019517.s004.doc]

**Table S2.** Linear mapping clustering results using different colour map sizes and up to 4 indices/cluster for each map size. Sequences highlighted in green starts a new cluster and are the centroids for clusters containing <= 2 sequences. Otherwise, centroids are highlighted in yellow

| **Hash Ranges** | **64** | | | | **128** | | | | **256** | | | | **512** | | | | **1024** | | | | **2048** | | | | **4096** | | | |
| --- | --- | --- | --- | --- | --- | --- | --- | --- | --- | --- | --- | --- | --- | --- | --- | --- | --- | --- | --- | --- | --- | --- | --- | --- | --- | --- | --- | --- |
|  | **Index/Cluster** | | | | **Index/Cluster** | | | | **Index/Cluster** | | | | **Index/Cluster** | | | | **Index/Cluster** | | | | **Index/Cluster** | | | | **Index/Cluster** | | | |
|  | **1** | **2** | **3** | **4** | **1** | **2** | **3** | **4** | **1** | **2** | **3** | **4** | **1** | **2** | **3** | **4** | **1** | **2** | **3** | **4** | **1** | **2** | **3** | **4** | **1** | **2** | **3** | **4** |
| **Strain** |  |  |  |  |  |  |  |  |  |  |  |  |  |  |  |  |  |  |  |  |  |  |  |  |  |  |  |  |
| N.seriolae-AB060281 | 1 | 1 | 1 | 1 | 1 | 1 | 1 | 1 | 1 | 1 | 1 | 1 | 1 | 1 | 1 | 1 | 1 | 1 | 1 | 1 | 1 | 1 | 1 | 1 | 1 | 1 | 1 | 1 |
| N.seriolae-AB060282 | 1 | 1 | 1 | 1 | 1 | 1 | 1 | 1 | 1 | 1 | 1 | 1 | 1 | 1 | 1 | 1 | 1 | 1 | 1 | 1 | 1 | 1 | 1 | 1 | 1 | 1 | 1 | 1 |
| N.asteroides-X53205 | 2 | 2 | 2 | 2 | 2 | 2 | 2 | 2 | 2 | 2 | 2 | 2 | 2 | 2 | 2 | 2 | 2 | 2 | 2 | 2 | 2 | 2 | 2 | 2 | 2 | 2 | 2 | 2 |
| N.globerula-AF430065 | 3 | 3 | 3 | 3 | 3 | 3 | 3 | 3 | 3 | 3 | 3 | 3 | 3 | 3 | 3 | 3 | 3 | 3 | 3 | 3 | 3 | 3 | 3 | 3 | 3 | 3 | 3 | 3 |
| N.globerula-DQ525592 | 3 | 3 | 3 | 3 | 3 | 3 | 3 | 3 | 3 | 3 | 3 | 3 | 3 | 3 | 3 | 3 | 3 | 3 | 3 | 3 | 3 | 3 | 3 | 3 | 3 | 3 | 3 | 3 |
| N.corynebacterioides-AF430066 | 4 | 4 | 4 | 3 | 4 | 4 | 4 | 4 | 4 | 4 | 4 | 4 | 4 | 4 | 4 | 4 | 4 | 4 | 4 | 4 | 4 | 4 | 4 | 4 | 4 | 4 | 4 | 4 |
| N.corynebacterioides-AY438619 | 4 | 4 | 4 | 3 | 4 | 4 | 4 | 4 | 4 | 4 | 4 | 4 | 4 | 4 | 4 | 4 | 4 | 4 | 4 | 4 | 4 | 4 | 4 | 4 | 4 | 4 | 4 | 4 |
| N.soli-AF277223 | 5 | 5 | 5 | 4 | 5 | 5 | 5 | 5 | 5 | 5 | 5 | 5 | 5 | 5 | 5 | 5 | 5 | 5 | 5 | 5 | 5 | 5 | 5 | 5 | 5 | 5 | 5 | 5 |
| N.coubleae-DQ235688 | 5 | 5 | 5 | 4 | 5 | 5 | 5 | 5 | 5 | 5 | 5 | 5 | 5 | 5 | 5 | 5 | 5 | 5 | 5 | 5 | 5 | 5 | 5 | 5 | 5 | 5 | 5 | 5 |
| N.ignorata-AJ303008 | 5 | 5 | 5 | 4 | 6 | 5 | 5 | 5 | 6 | 6 | 5 | 5 | 6 | 6 | 6 | 6 | 6 | 6 | 6 | 6 | 6 | 6 | 6 | 6 | 6 | 6 | 6 | 6 |
| N.ignorata-AY191254 | 5 | 5 | 5 | 4 | 6 | 5 | 5 | 5 | 6 | 6 | 5 | 5 | 6 | 6 | 6 | 6 | 6 | 6 | 6 | 6 | 6 | 6 | 6 | 6 | 6 | 6 | 6 | 6 |
| N.ignorata-DQ659907 | 5 | 5 | 5 | 4 | 6 | 5 | 5 | 5 | 6 | 6 | 5 | 5 | 6 | 6 | 6 | 6 | 6 | 6 | 6 | 6 | 6 | 6 | 6 | 6 | 6 | 6 | 6 | 6 |
| N.soli-AF277199 | 5 | 5 | 5 | 4 | 7 | 5 | 5 | 5 | 7 | 7 | 6 | 5 | 7 | 7 | 7 | 7 | 7 | 7 | 7 | 7 | 7 | 7 | 7 | 7 | 7 | 7 | 7 | 7 |
| N.soli-AF430051 | 5 | 5 | 5 | 4 | 7 | 5 | 5 | 5 | 7 | 7 | 6 | 5 | 7 | 7 | 7 | 7 | 8 | 7 | 7 | 7 | 8 | 8 | 8 | 7 | 8 | 8 | 8 | 8 |
| N.cummidelens-AF277202 | 5 | 5 | 5 | 4 | 7 | 5 | 5 | 5 | 7 | 7 | 6 | 5 | 7 | 7 | 7 | 7 | 8 | 7 | 7 | 7 | 8 | 8 | 8 | 7 | 8 | 8 | 8 | 8 |
| N.salmonicida-Z46750 | 5 | 5 | 5 | 4 | 8 | 5 | 5 | 5 | 8 | 8 | 6 | 5 | 8 | 8 | 8 | 8 | 9 | 8 | 8 | 8 | 9 | 9 | 9 | 8 | 9 | 9 | 9 | 9 |
| N.salmonicida-AF430050 | 5 | 5 | 5 | 4 | 8 | 5 | 5 | 5 | 8 | 8 | 6 | 5 | 8 | 8 | 8 | 8 | 9 | 8 | 8 | 8 | 9 | 9 | 9 | 8 | 9 | 9 | 9 | 9 |
| N.fluminea-AF277204 | 6 | 5 | 5 | 4 | 9 | 6 | 5 | 5 | 9 | 9 | 7 | 6 | 9 | 9 | 9 | 9 | 10 | 9 | 9 | 9 | 10 | 10 | 10 | 9 | 10 | 10 | 10 | 10 |
| N.fluminea-AF430053 | 6 | 5 | 5 | 4 | 9 | 6 | 5 | 5 | 9 | 9 | 7 | 6 | 9 | 9 | 9 | 9 | 10 | 9 | 9 | 9 | 10 | 10 | 10 | 9 | 10 | 10 | 10 | 10 |
| N.pigrifrangens-AF219974 | 7 | 6 | 6 | 5 | 10 | 7 | 6 | 6 | 10 | 10 | 8 | 7 | 10 | 10 | 10 | 10 | 10 | 10 | 10 | 10 | 10 | 10 | 10 | 10 | 10 | 10 | 10 | 10 |
| N.jejuensis-AY964666 | 8 | 7 | 7 | 5 | 11 | 8 | 7 | 7 | 11 | 11 | 9 | 8 | 11 | 11 | 11 | 11 | 11 | 11 | 11 | 11 | 11 | 11 | 11 | 11 | 11 | 11 | 11 | 11 |
| N.alba-EU249584 | 8 | 7 | 7 | 5 | 11 | 8 | 7 | 7 | 11 | 11 | 9 | 8 | 11 | 11 | 11 | 11 | 12 | 11 | 11 | 11 | 12 | 12 | 12 | 11 | 12 | 12 | 12 | 12 |
| N.ninae-DQ235687 | 8 | 7 | 7 | 5 | 12 | 8 | 7 | 7 | 12 | 12 | 9 | 8 | 12 | 12 | 12 | 12 | 12 | 12 | 12 | 12 | 12 | 12 | 12 | 12 | 12 | 12 | 12 | 12 |
| N.alba-AY222321 | 8 | 7 | 7 | 5 | 12 | 8 | 7 | 7 | 12 | 12 | 9 | 8 | 12 | 12 | 12 | 12 | 12 | 12 | 12 | 12 | 12 | 12 | 12 | 12 | 12 | 12 | 12 | 12 |
| N.mexicana-AY555577 | 9 | 8 | 7 | 5 | 13 | 9 | 8 | 8 | 13 | 13 | 10 | 9 | 13 | 13 | 13 | 13 | 13 | 13 | 13 | 13 | 13 | 13 | 13 | 13 | 13 | 13 | 13 | 13 |
| N.mexicana-AY560655 | 9 | 8 | 7 | 5 | 13 | 9 | 8 | 8 | 13 | 13 | 10 | 9 | 13 | 13 | 13 | 13 | 13 | 13 | 13 | 13 | 13 | 13 | 13 | 13 | 13 | 13 | 13 | 13 |
| N.caishijiensis-AF459443 | 10 | 8 | 8 | 5 | 13 | 10 | 9 | 8 | 13 | 13 | 11 | 10 | 13 | 13 | 13 | 13 | 14 | 13 | 13 | 13 | 14 | 14 | 14 | 13 | 14 | 14 | 14 | 14 |
| N.carnea-X80602 | 11 | 9 | 9 | 5 | 14 | 11 | 10 | 9 | 14 | 14 | 12 | 11 | 14 | 14 | 14 | 14 | 15 | 14 | 14 | 14 | 15 | 15 | 15 | 14 | 15 | 15 | 15 | 15 |
| N.carnea-AF430035 | 11 | 9 | 9 | 5 | 14 | 11 | 10 | 9 | 14 | 14 | 12 | 11 | 15 | 14 | 14 | 14 | 15 | 15 | 14 | 14 | 15 | 15 | 15 | 15 | 15 | 15 | 15 | 15 |
| N.carnea-X80607 | 11 | 9 | 9 | 5 | 14 | 11 | 10 | 9 | 14 | 14 | 12 | 11 | 15 | 14 | 14 | 14 | 15 | 15 | 14 | 14 | 15 | 15 | 15 | 15 | 15 | 15 | 15 | 15 |
| N.carnea-Z36929 | 11 | 9 | 9 | 5 | 14 | 11 | 10 | 9 | 14 | 14 | 12 | 11 | 15 | 14 | 14 | 14 | 15 | 15 | 14 | 14 | 15 | 15 | 15 | 15 | 15 | 15 | 15 | 15 |
| N.carnea-AF430036 | 11 | 9 | 9 | 5 | 14 | 11 | 10 | 9 | 14 | 14 | 12 | 11 | 16 | 14 | 14 | 14 | 16 | 16 | 14 | 14 | 16 | 16 | 16 | 16 | 16 | 16 | 16 | 16 |
| N.carnea-AF430037 | 11 | 9 | 9 | 5 | 14 | 11 | 10 | 9 | 14 | 14 | 12 | 11 | 16 | 14 | 14 | 14 | 16 | 16 | 14 | 14 | 16 | 16 | 16 | 16 | 16 | 16 | 16 | 16 |
| N.testacea-AB121769 | 12 | 9 | 9 | 5 | 15 | 12 | 10 | 9 | 15 | 15 | 13 | 12 | 17 | 15 | 15 | 15 | 17 | 17 | 15 | 15 | 17 | 17 | 17 | 17 | 17 | 17 | 17 | 17 |
| N.sienata-AB121770 | 12 | 9 | 9 | 5 | 15 | 12 | 10 | 9 | 16 | 15 | 13 | 12 | 18 | 16 | 15 | 15 | 18 | 18 | 15 | 15 | 18 | 18 | 18 | 18 | 18 | 18 | 18 | 18 |
| N.testacea-AB192415 | 12 | 9 | 9 | 5 | 15 | 12 | 10 | 9 | 16 | 15 | 13 | 12 | 18 | 16 | 15 | 15 | 18 | 18 | 15 | 15 | 18 | 18 | 18 | 18 | 18 | 18 | 18 | 18 |
| N.flavorosea-AF430048 | 12 | 9 | 9 | 5 | 16 | 12 | 10 | 9 | 17 | 16 | 13 | 12 | 19 | 17 | 16 | 16 | 19 | 19 | 16 | 16 | 19 | 19 | 19 | 19 | 19 | 19 | 19 | 19 |
| N.flavorosea-Z46754 | 12 | 9 | 9 | 5 | 16 | 12 | 10 | 9 | 17 | 16 | 13 | 12 | 19 | 17 | 16 | 16 | 19 | 19 | 16 | 16 | 19 | 19 | 19 | 19 | 19 | 19 | 19 | 19 |
| N.asteroides-AF163818 | 12 | 9 | 9 | 5 | 17 | 12 | 10 | 9 | 18 | 17 | 13 | 12 | 20 | 18 | 17 | 17 | 20 | 20 | 17 | 17 | 20 | 20 | 20 | 20 | 20 | 20 | 20 | 20 |
| N.asteroides-Z82230 | 12 | 9 | 9 | 5 | 17 | 12 | 10 | 9 | 18 | 17 | 13 | 12 | 20 | 18 | 17 | 17 | 20 | 20 | 17 | 17 | 20 | 20 | 20 | 20 | 20 | 20 | 20 | 20 |
| N.asteroides-Z82231 | 13 | 9 | 9 | 5 | 18 | 13 | 10 | 9 | 19 | 18 | 14 | 13 | 21 | 19 | 18 | 18 | 21 | 21 | 18 | 18 | 21 | 21 | 21 | 21 | 21 | 21 | 21 | 21 |
| N.jinanensis-DQ462650 | 13 | 9 | 9 | 5 | 18 | 13 | 10 | 9 | 19 | 18 | 14 | 13 | 21 | 19 | 18 | 18 | 21 | 21 | 18 | 18 | 21 | 21 | 21 | 21 | 21 | 21 | 21 | 21 |
| N.speluncae-AM422449 | 13 | 9 | 9 | 5 | 18 | 13 | 10 | 9 | 19 | 18 | 14 | 13 | 21 | 19 | 18 | 18 | 21 | 21 | 18 | 18 | 21 | 21 | 21 | 21 | 21 | 21 | 21 | 21 |
| N.tenerifensis-AJ556157 | 14 | 10 | 9 | 5 | 19 | 14 | 11 | 10 | 20 | 19 | 15 | 14 | 22 | 20 | 19 | 19 | 22 | 22 | 19 | 19 | 22 | 22 | 22 | 22 | 22 | 22 | 22 | 22 |
| N.altamirensis-EU006090 | 14 | 10 | 9 | 5 | 19 | 14 | 11 | 10 | 20 | 19 | 15 | 14 | 22 | 20 | 19 | 19 | 22 | 22 | 19 | 19 | 22 | 22 | 22 | 22 | 22 | 22 | 22 | 22 |
| N.iowensis-DQ925490 | 15 | 10 | 9 | 5 | 20 | 14 | 11 | 10 | 21 | 20 | 15 | 14 | 23 | 21 | 20 | 20 | 23 | 23 | 20 | 20 | 23 | 23 | 23 | 23 | 23 | 23 | 23 | 23 |
| N.brasiliensis-X80591 | 15 | 10 | 9 | 5 | 20 | 14 | 11 | 10 | 22 | 20 | 16 | 14 | 24 | 22 | 20 | 20 | 24 | 24 | 20 | 20 | 24 | 24 | 24 | 24 | 24 | 24 | 24 | 24 |
| N.brasiliensis-AY245543 | 15 | 10 | 9 | 5 | 20 | 14 | 11 | 10 | 22 | 20 | 16 | 14 | 24 | 22 | 20 | 20 | 24 | 24 | 20 | 20 | 24 | 24 | 24 | 24 | 24 | 24 | 24 | 24 |
| N.brasiliensis-Z36935 | 15 | 10 | 9 | 5 | 20 | 14 | 11 | 10 | 22 | 20 | 16 | 14 | 24 | 22 | 20 | 20 | 24 | 24 | 20 | 20 | 25 | 24 | 24 | 24 | 25 | 25 | 25 | 24 |
| N.brasiliensis-AF430038 | 15 | 10 | 9 | 5 | 20 | 14 | 11 | 10 | 22 | 20 | 16 | 14 | 24 | 22 | 20 | 20 | 24 | 24 | 20 | 20 | 25 | 24 | 24 | 24 | 25 | 25 | 25 | 24 |
| N.brasiliensis-X80608 | 15 | 10 | 9 | 5 | 20 | 14 | 11 | 10 | 22 | 20 | 16 | 14 | 24 | 22 | 20 | 20 | 24 | 24 | 20 | 20 | 26 | 24 | 24 | 24 | 26 | 26 | 26 | 24 |
| N.brasiliensis-DQ659902 | 15 | 10 | 9 | 5 | 20 | 14 | 11 | 10 | 22 | 20 | 16 | 14 | 24 | 22 | 20 | 20 | 24 | 24 | 20 | 20 | 26 | 24 | 24 | 24 | 26 | 26 | 26 | 24 |
| N.brevicatena-Z36928 | 16 | 11 | 10 | 5 | 21 | 15 | 12 | 11 | 23 | 21 | 17 | 15 | 25 | 23 | 21 | 21 | 25 | 25 | 21 | 21 | 27 | 25 | 25 | 25 | 27 | 27 | 27 | 25 |
| N.brevicatena-X80600 | 16 | 11 | 10 | 5 | 21 | 15 | 12 | 11 | 24 | 21 | 17 | 15 | 26 | 24 | 22 | 21 | 26 | 26 | 22 | 21 | 27 | 26 | 26 | 26 | 27 | 27 | 27 | 26 |
| N.brevicatena-AF430040 | 16 | 11 | 10 | 5 | 21 | 15 | 12 | 11 | 24 | 21 | 17 | 15 | 26 | 24 | 22 | 21 | 26 | 26 | 22 | 21 | 27 | 26 | 26 | 26 | 27 | 27 | 27 | 26 |
| N.brevicatena-DQ659903 | 16 | 11 | 10 | 5 | 21 | 15 | 12 | 11 | 24 | 21 | 17 | 15 | 26 | 24 | 22 | 21 | 26 | 26 | 22 | 21 | 27 | 26 | 26 | 26 | 27 | 27 | 27 | 26 |
| N.paucivorans-AJ437308 | 16 | 11 | 10 | 5 | 22 | 15 | 12 | 11 | 25 | 22 | 17 | 15 | 27 | 25 | 23 | 22 | 27 | 27 | 23 | 22 | 28 | 27 | 27 | 27 | 28 | 28 | 28 | 27 |
| N.paucivorans-AF430041 | 16 | 11 | 10 | 5 | 22 | 15 | 12 | 11 | 25 | 22 | 17 | 15 | 28 | 25 | 23 | 22 | 28 | 28 | 23 | 22 | 29 | 28 | 28 | 28 | 29 | 29 | 29 | 28 |
| N.paucivorans-AF179865 | 16 | 11 | 10 | 5 | 22 | 15 | 12 | 11 | 25 | 22 | 17 | 15 | 28 | 25 | 23 | 22 | 28 | 28 | 23 | 22 | 29 | 28 | 28 | 28 | 29 | 29 | 29 | 28 |
| N.paucivorans-DQ659913 | 16 | 11 | 10 | 5 | 22 | 15 | 12 | 11 | 25 | 22 | 17 | 15 | 28 | 25 | 23 | 22 | 28 | 28 | 23 | 22 | 29 | 28 | 28 | 28 | 29 | 29 | 29 | 28 |
| N.puris-AB097453 | 17 | 12 | 10 | 5 | 23 | 16 | 13 | 12 | 26 | 23 | 18 | 16 | 29 | 26 | 24 | 23 | 29 | 29 | 24 | 23 | 30 | 29 | 29 | 29 | 30 | 30 | 30 | 29 |
| N.puris-AB097454 | 17 | 12 | 10 | 5 | 23 | 16 | 13 | 12 | 26 | 23 | 18 | 16 | 29 | 26 | 24 | 23 | 29 | 29 | 24 | 23 | 30 | 29 | 29 | 29 | 30 | 30 | 30 | 29 |
| N.puris-AJ508748 | 17 | 12 | 10 | 5 | 23 | 16 | 13 | 12 | 27 | 23 | 18 | 16 | 30 | 27 | 24 | 23 | 30 | 30 | 24 | 23 | 31 | 30 | 30 | 30 | 31 | 31 | 31 | 30 |
| N.puris-AB097455 | 17 | 12 | 10 | 5 | 23 | 16 | 13 | 12 | 27 | 23 | 18 | 16 | 30 | 27 | 24 | 23 | 30 | 30 | 24 | 23 | 31 | 30 | 30 | 30 | 31 | 31 | 31 | 30 |
| N.takedensis-AB158277 | 18 | 13 | 10 | 5 | 24 | 17 | 14 | 13 | 28 | 24 | 19 | 17 | 31 | 28 | 25 | 24 | 31 | 31 | 25 | 24 | 32 | 31 | 31 | 31 | 32 | 32 | 32 | 31 |
| N.takedensis-AB158278 | 18 | 13 | 10 | 5 | 24 | 17 | 14 | 13 | 28 | 24 | 19 | 17 | 32 | 28 | 25 | 24 | 32 | 32 | 25 | 24 | 33 | 32 | 32 | 32 | 33 | 33 | 33 | 32 |
| N.takedensis-DQ840025 | 18 | 13 | 10 | 5 | 24 | 17 | 14 | 13 | 28 | 24 | 19 | 17 | 32 | 28 | 25 | 24 | 32 | 32 | 25 | 24 | 33 | 32 | 32 | 32 | 33 | 33 | 33 | 32 |
| N.amamiensis-AB275164 | 19 | 14 | 10 | 5 | 25 | 18 | 15 | 14 | 29 | 25 | 20 | 18 | 32 | 29 | 26 | 25 | 32 | 32 | 26 | 25 | 33 | 32 | 32 | 32 | 33 | 33 | 33 | 32 |
| N.thailandica-AB126874 | 20 | 14 | 10 | 5 | 26 | 19 | 15 | 14 | 30 | 26 | 21 | 19 | 33 | 30 | 27 | 26 | 33 | 33 | 27 | 26 | 34 | 33 | 33 | 33 | 34 | 34 | 34 | 33 |
| N.neocaledoniensis-AY282603 | 20 | 14 | 10 | 5 | 26 | 19 | 15 | 14 | 30 | 26 | 21 | 19 | 34 | 30 | 27 | 26 | 34 | 34 | 27 | 26 | 35 | 34 | 34 | 34 | 35 | 35 | 35 | 34 |
| N.asteroides-AF430025 | 21 | 14 | 10 | 5 | 27 | 19 | 15 | 14 | 31 | 27 | 22 | 19 | 34 | 31 | 28 | 27 | 34 | 34 | 28 | 27 | 35 | 34 | 34 | 34 | 35 | 35 | 35 | 34 |
| N.asteroides-AF430026 | 21 | 14 | 10 | 5 | 27 | 19 | 15 | 14 | 31 | 27 | 22 | 19 | 34 | 31 | 28 | 27 | 34 | 34 | 28 | 27 | 35 | 34 | 34 | 34 | 35 | 35 | 35 | 34 |
| N.asteroides-X84850 | 21 | 14 | 10 | 5 | 28 | 19 | 15 | 14 | 32 | 28 | 22 | 19 | 35 | 32 | 29 | 28 | 35 | 35 | 29 | 28 | 36 | 35 | 35 | 35 | 36 | 36 | 36 | 35 |
| N.asteroides-X80606 | 21 | 14 | 10 | 5 | 28 | 19 | 15 | 14 | 32 | 28 | 22 | 19 | 35 | 32 | 29 | 28 | 35 | 35 | 29 | 28 | 36 | 35 | 35 | 35 | 36 | 36 | 36 | 35 |
| N.asteroides-Z36934 | 21 | 14 | 10 | 5 | 28 | 19 | 15 | 14 | 32 | 28 | 22 | 19 | 35 | 32 | 29 | 28 | 35 | 35 | 29 | 28 | 36 | 35 | 35 | 35 | 36 | 36 | 36 | 35 |
| N.asteroides-AF430019 | 21 | 14 | 10 | 5 | 28 | 19 | 15 | 14 | 32 | 28 | 22 | 19 | 35 | 32 | 29 | 28 | 36 | 35 | 29 | 28 | 37 | 36 | 35 | 35 | 37 | 37 | 37 | 36 |
| N.nova-AB292584 | 21 | 14 | 10 | 5 | 28 | 19 | 15 | 14 | 32 | 28 | 22 | 19 | 35 | 32 | 29 | 28 | 36 | 35 | 29 | 28 | 37 | 36 | 35 | 35 | 37 | 37 | 37 | 36 |
| N.asteroides-DQ659898 | 21 | 14 | 10 | 5 | 28 | 19 | 15 | 14 | 32 | 28 | 22 | 19 | 35 | 32 | 29 | 28 | 36 | 35 | 29 | 28 | 37 | 36 | 35 | 35 | 37 | 37 | 37 | 36 |
| N.cyriacigeorgica-AB094576 | 22 | 15 | 10 | 5 | 29 | 20 | 16 | 15 | 33 | 29 | 23 | 20 | 36 | 33 | 30 | 29 | 37 | 36 | 30 | 29 | 38 | 37 | 36 | 36 | 38 | 38 | 38 | 37 |
| N.asteroides-X57949 | 22 | 15 | 10 | 5 | 30 | 20 | 16 | 15 | 34 | 30 | 24 | 20 | 37 | 34 | 31 | 30 | 38 | 37 | 31 | 30 | 39 | 38 | 37 | 37 | 39 | 39 | 39 | 38 |
| N.asteroides-Z82218 | 22 | 15 | 10 | 5 | 30 | 20 | 16 | 15 | 34 | 30 | 24 | 20 | 37 | 34 | 31 | 30 | 38 | 37 | 31 | 30 | 39 | 38 | 37 | 37 | 39 | 39 | 39 | 38 |
| N.cyriacigeorgica-DQ267485 | 22 | 15 | 10 | 5 | 30 | 20 | 16 | 15 | 34 | 30 | 24 | 20 | 37 | 34 | 31 | 30 | 38 | 37 | 31 | 30 | 39 | 38 | 37 | 37 | 39 | 39 | 39 | 38 |
| N.cyriacigeorgica-AB094570 | 22 | 15 | 10 | 5 | 30 | 20 | 16 | 15 | 34 | 30 | 24 | 20 | 37 | 34 | 31 | 30 | 38 | 37 | 31 | 30 | 40 | 38 | 37 | 37 | 40 | 40 | 40 | 38 |
| N.cyriacigeorgica-EF127500 | 22 | 15 | 10 | 5 | 30 | 20 | 16 | 15 | 34 | 30 | 24 | 20 | 37 | 34 | 31 | 30 | 38 | 37 | 31 | 30 | 40 | 38 | 37 | 37 | 40 | 40 | 40 | 38 |
| N.cyriacigeorgica-AB115948 | 22 | 15 | 10 | 5 | 31 | 20 | 16 | 15 | 35 | 31 | 24 | 20 | 38 | 35 | 32 | 30 | 39 | 38 | 32 | 30 | 41 | 39 | 38 | 38 | 41 | 41 | 41 | 39 |
| N.cyriacigeorgica-AB094581 | 22 | 15 | 10 | 5 | 31 | 20 | 16 | 15 | 36 | 31 | 24 | 20 | 39 | 36 | 33 | 30 | 40 | 39 | 33 | 30 | 42 | 40 | 39 | 39 | 42 | 42 | 42 | 40 |
| N.cyriacigeorgica-AB094579 | 22 | 15 | 10 | 5 | 31 | 20 | 16 | 15 | 36 | 31 | 24 | 20 | 39 | 36 | 33 | 30 | 40 | 39 | 33 | 30 | 42 | 40 | 39 | 39 | 42 | 42 | 42 | 40 |
| N.cyriacigeorgica-AJ508414 | 22 | 15 | 10 | 5 | 31 | 20 | 16 | 15 | 36 | 31 | 24 | 20 | 39 | 36 | 33 | 30 | 40 | 39 | 33 | 30 | 42 | 40 | 39 | 39 | 42 | 42 | 42 | 40 |
| N.cyriacigeorgica-AB094569 | 22 | 15 | 10 | 5 | 31 | 20 | 16 | 15 | 36 | 31 | 24 | 20 | 39 | 36 | 33 | 30 | 40 | 39 | 33 | 30 | 42 | 40 | 39 | 39 | 42 | 42 | 42 | 40 |
| N.cyriacigeorgica-AB094568 | 22 | 15 | 10 | 5 | 31 | 20 | 16 | 15 | 36 | 31 | 24 | 20 | 39 | 36 | 33 | 30 | 40 | 39 | 33 | 30 | 42 | 40 | 39 | 39 | 42 | 42 | 42 | 40 |
| N.cyriacigeorgica-AB094565 | 22 | 15 | 10 | 5 | 31 | 20 | 16 | 15 | 36 | 31 | 24 | 20 | 39 | 36 | 33 | 30 | 40 | 39 | 33 | 30 | 42 | 40 | 39 | 39 | 42 | 42 | 42 | 40 |
| Ncyriacigeorgica-AB094566 | 22 | 15 | 10 | 5 | 31 | 20 | 16 | 15 | 36 | 31 | 24 | 20 | 40 | 36 | 33 | 30 | 41 | 40 | 33 | 30 | 43 | 41 | 40 | 40 | 43 | 43 | 43 | 41 |
| N.cyriacigeorgica-AB094567 | 22 | 15 | 10 | 5 | 31 | 20 | 16 | 15 | 36 | 31 | 24 | 20 | 40 | 36 | 33 | 30 | 42 | 40 | 33 | 30 | 44 | 42 | 40 | 40 | 44 | 44 | 44 | 42 |
| N.cyriacigeorgica-AB094571 | 22 | 15 | 10 | 5 | 31 | 20 | 16 | 15 | 36 | 31 | 24 | 20 | 40 | 36 | 33 | 30 | 42 | 40 | 33 | 30 | 44 | 42 | 40 | 40 | 44 | 44 | 44 | 42 |
| N.cyriacigeorgica-AB094573 | 22 | 15 | 10 | 5 | 31 | 20 | 16 | 15 | 36 | 31 | 24 | 20 | 40 | 36 | 33 | 30 | 42 | 40 | 33 | 30 | 45 | 42 | 40 | 40 | 45 | 45 | 45 | 42 |
| N.cyriacigeorgica-AB094574 | 22 | 15 | 10 | 5 | 31 | 20 | 16 | 15 | 36 | 31 | 24 | 20 | 40 | 36 | 33 | 30 | 42 | 40 | 33 | 30 | 45 | 42 | 40 | 40 | 45 | 45 | 45 | 42 |
| N.cyriacigeorgica-AB094578 | 22 | 15 | 10 | 5 | 31 | 20 | 16 | 15 | 36 | 31 | 24 | 20 | 40 | 36 | 33 | 30 | 42 | 40 | 33 | 30 | 45 | 42 | 40 | 40 | 45 | 45 | 45 | 42 |
| N.cyriacigeorgica-AB094580 | 22 | 15 | 10 | 5 | 31 | 20 | 16 | 15 | 36 | 31 | 24 | 20 | 40 | 36 | 33 | 30 | 42 | 40 | 33 | 30 | 46 | 42 | 40 | 40 | 46 | 46 | 46 | 42 |
| N.cyriacigeorgica-AB094582 | 22 | 15 | 10 | 5 | 31 | 20 | 16 | 15 | 37 | 31 | 24 | 20 | 41 | 37 | 33 | 30 | 43 | 41 | 33 | 30 | 47 | 43 | 41 | 41 | 47 | 47 | 47 | 43 |
| N.cyriacigeorgica-AB115949 | 22 | 15 | 10 | 5 | 31 | 20 | 16 | 15 | 37 | 31 | 24 | 20 | 42 | 37 | 33 | 30 | 44 | 42 | 33 | 30 | 47 | 44 | 42 | 42 | 47 | 47 | 47 | 44 |
| N.cyriacigeorgica-AB115950 | 22 | 15 | 10 | 5 | 31 | 20 | 16 | 15 | 37 | 31 | 24 | 20 | 42 | 37 | 33 | 30 | 44 | 42 | 33 | 30 | 47 | 44 | 42 | 42 | 47 | 47 | 47 | 44 |
| N.cyriacigeorgica-AB115952 | 22 | 15 | 10 | 5 | 31 | 20 | 16 | 15 | 38 | 31 | 24 | 20 | 43 | 38 | 33 | 30 | 45 | 43 | 33 | 30 | 48 | 45 | 43 | 43 | 48 | 48 | 48 | 45 |
| N.cyriacigeorgica-AB115953 | 22 | 15 | 10 | 5 | 31 | 20 | 16 | 15 | 38 | 31 | 24 | 20 | 43 | 38 | 33 | 30 | 45 | 43 | 33 | 30 | 48 | 45 | 43 | 43 | 48 | 48 | 48 | 45 |
| N.cyriacigeorgica-AB115954 | 22 | 15 | 10 | 5 | 31 | 20 | 16 | 15 | 39 | 31 | 24 | 20 | 44 | 39 | 33 | 30 | 46 | 44 | 33 | 30 | 49 | 46 | 44 | 44 | 49 | 49 | 49 | 46 |
| N.cyriacigeorgica-AB115955 | 22 | 15 | 10 | 5 | 31 | 20 | 16 | 15 | 39 | 31 | 24 | 20 | 44 | 39 | 33 | 30 | 46 | 44 | 33 | 30 | 50 | 46 | 44 | 44 | 50 | 50 | 50 | 46 |
| N.cyriacigeorgica-AF282889 | 22 | 15 | 10 | 5 | 31 | 20 | 16 | 15 | 39 | 31 | 24 | 20 | 44 | 39 | 33 | 30 | 46 | 44 | 33 | 30 | 50 | 46 | 44 | 44 | 50 | 50 | 50 | 46 |
| N.cyriacigeorgica-AF430020 | 22 | 15 | 10 | 5 | 31 | 20 | 16 | 15 | 39 | 31 | 24 | 20 | 44 | 39 | 33 | 30 | 46 | 44 | 33 | 30 | 50 | 46 | 44 | 44 | 50 | 50 | 50 | 46 |
| N.cyriacigeorgica-AF430027 | 22 | 15 | 10 | 5 | 31 | 20 | 16 | 15 | 39 | 31 | 24 | 20 | 44 | 39 | 33 | 30 | 46 | 44 | 33 | 30 | 51 | 46 | 44 | 44 | 51 | 51 | 51 | 46 |
| N.cyriacigeorgica-AY244782 | 22 | 15 | 10 | 5 | 31 | 20 | 16 | 15 | 39 | 31 | 24 | 20 | 44 | 39 | 33 | 30 | 46 | 44 | 33 | 30 | 51 | 46 | 44 | 44 | 51 | 51 | 51 | 46 |
| N.cyriacigeorgica-DQ303128 | 22 | 15 | 10 | 5 | 31 | 20 | 16 | 15 | 39 | 31 | 24 | 20 | 44 | 39 | 33 | 30 | 47 | 44 | 33 | 30 | 52 | 47 | 44 | 44 | 52 | 52 | 52 | 47 |
| N.cyriacigeorgica-EF127498 | 22 | 15 | 10 | 5 | 31 | 20 | 16 | 15 | 39 | 31 | 24 | 20 | 44 | 39 | 33 | 30 | 47 | 44 | 33 | 30 | 52 | 47 | 44 | 44 | 52 | 52 | 52 | 47 |
| N.cyriacigeorgica-EF127499 | 22 | 15 | 10 | 5 | 31 | 20 | 16 | 15 | 39 | 31 | 24 | 20 | 44 | 39 | 33 | 30 | 48 | 44 | 33 | 30 | 52 | 48 | 45 | 44 | 52 | 52 | 52 | 48 |
| N.cyriacigeorgica-EF127501 | 22 | 15 | 10 | 5 | 31 | 20 | 16 | 15 | 39 | 31 | 24 | 20 | 44 | 39 | 33 | 30 | 49 | 44 | 33 | 30 | 53 | 49 | 45 | 44 | 53 | 53 | 53 | 49 |
| N.cyriacigeorgica-EF127502 | 22 | 15 | 10 | 5 | 31 | 20 | 16 | 15 | 39 | 31 | 24 | 20 | 44 | 39 | 33 | 30 | 49 | 44 | 33 | 30 | 53 | 49 | 45 | 44 | 53 | 53 | 53 | 49 |
| N.asteroides-DQ659900 | 22 | 15 | 10 | 5 | 31 | 20 | 16 | 15 | 39 | 31 | 24 | 20 | 44 | 39 | 33 | 30 | 49 | 44 | 33 | 30 | 53 | 49 | 45 | 44 | 53 | 53 | 53 | 49 |
| N.asteroides-AF162772 | 22 | 15 | 10 | 5 | 31 | 20 | 16 | 15 | 39 | 31 | 24 | 20 | 44 | 39 | 33 | 30 | 49 | 44 | 33 | 30 | 53 | 49 | 45 | 44 | 53 | 53 | 53 | 49 |
| N.cyriacigeorgica-AB094577 | 22 | 15 | 10 | 5 | 31 | 20 | 16 | 15 | 40 | 31 | 24 | 20 | 45 | 40 | 34 | 30 | 50 | 45 | 34 | 30 | 54 | 50 | 46 | 45 | 54 | 54 | 54 | 50 |
| N.cyriacigeorgica-AB115951 | 22 | 15 | 10 | 5 | 31 | 20 | 16 | 15 | 41 | 31 | 24 | 20 | 46 | 41 | 35 | 30 | 51 | 46 | 35 | 30 | 55 | 51 | 47 | 46 | 55 | 55 | 55 | 51 |
| N.cyriacigeorgica-AB094585 | 22 | 15 | 10 | 5 | 31 | 20 | 16 | 15 | 41 | 31 | 24 | 20 | 46 | 41 | 35 | 30 | 51 | 46 | 35 | 30 | 55 | 51 | 47 | 46 | 55 | 55 | 55 | 51 |
| N.cyriacigeorgica-AB094575 | 22 | 15 | 10 | 5 | 31 | 20 | 16 | 15 | 41 | 31 | 24 | 20 | 46 | 41 | 35 | 30 | 52 | 46 | 35 | 30 | 56 | 52 | 47 | 46 | 56 | 56 | 56 | 52 |
| N.cyriacigeorgica-AB094572 | 22 | 15 | 10 | 5 | 31 | 20 | 16 | 15 | 41 | 31 | 24 | 20 | 46 | 41 | 35 | 30 | 52 | 46 | 35 | 30 | 56 | 52 | 47 | 46 | 56 | 56 | 56 | 52 |
| N.cyriacigeorgica-AB094584 | 22 | 15 | 10 | 5 | 31 | 20 | 16 | 15 | 41 | 31 | 24 | 20 | 46 | 41 | 35 | 30 | 52 | 46 | 35 | 30 | 56 | 52 | 47 | 46 | 56 | 56 | 56 | 52 |
| N.cyriacigeorgica-DQ659904 | 22 | 15 | 10 | 5 | 31 | 20 | 16 | 15 | 41 | 31 | 24 | 20 | 46 | 41 | 35 | 30 | 52 | 46 | 35 | 30 | 56 | 52 | 47 | 46 | 56 | 56 | 56 | 52 |
| N.cyriacigeorgica-AB094583 | 22 | 15 | 10 | 5 | 31 | 20 | 16 | 15 | 41 | 31 | 24 | 20 | 46 | 41 | 35 | 30 | 52 | 46 | 35 | 30 | 56 | 52 | 47 | 46 | 56 | 56 | 56 | 52 |
| N.pneumoniae-AB108780 | 23 | 16 | 11 | 5 | 32 | 21 | 17 | 16 | 42 | 32 | 25 | 21 | 47 | 42 | 36 | 31 | 53 | 47 | 36 | 31 | 57 | 53 | 48 | 47 | 57 | 57 | 57 | 53 |
| N.polyresistens-AY626158 | 24 | 17 | 12 | 5 | 33 | 22 | 18 | 17 | 43 | 33 | 26 | 22 | 48 | 43 | 37 | 32 | 54 | 48 | 37 | 32 | 58 | 54 | 49 | 48 | 58 | 58 | 58 | 54 |
| Nlijiangensis-AY779043 | 24 | 17 | 12 | 5 | 33 | 22 | 18 | 17 | 43 | 33 | 26 | 22 | 48 | 43 | 37 | 32 | 54 | 48 | 37 | 32 | 58 | 54 | 49 | 48 | 58 | 58 | 58 | 54 |
| N.xishanensis-AY333115 | 24 | 17 | 12 | 5 | 33 | 22 | 18 | 17 | 43 | 33 | 26 | 22 | 48 | 43 | 37 | 32 | 54 | 48 | 37 | 32 | 58 | 54 | 49 | 48 | 58 | 58 | 58 | 54 |
| N.higoensis-AB108778 | 25 | 18 | 12 | 5 | 34 | 23 | 19 | 18 | 44 | 34 | 27 | 23 | 49 | 44 | 38 | 33 | 55 | 49 | 38 | 33 | 59 | 55 | 50 | 49 | 59 | 59 | 59 | 55 |
| N.shimofusensis-AB108777 | 25 | 18 | 12 | 5 | 35 | 23 | 19 | 18 | 45 | 35 | 27 | 23 | 50 | 45 | 39 | 34 | 56 | 50 | 39 | 34 | 60 | 56 | 51 | 50 | 60 | 60 | 60 | 56 |
| N.shimofusensis-AB108775 | 25 | 18 | 12 | 5 | 35 | 23 | 19 | 18 | 45 | 35 | 27 | 23 | 50 | 45 | 39 | 34 | 56 | 50 | 39 | 34 | 60 | 56 | 51 | 50 | 60 | 60 | 60 | 56 |
| N.shimofusensis-AB108776 | 25 | 18 | 12 | 5 | 35 | 23 | 19 | 18 | 45 | 35 | 27 | 23 | 50 | 45 | 39 | 34 | 56 | 50 | 39 | 34 | 60 | 56 | 51 | 50 | 60 | 60 | 60 | 56 |
| N.otitidiscaviarum-AF475084 | 25 | 18 | 12 | 5 | 36 | 23 | 19 | 18 | 46 | 36 | 27 | 23 | 51 | 46 | 40 | 35 | 57 | 51 | 40 | 35 | 61 | 57 | 52 | 51 | 61 | 61 | 61 | 57 |
| Nfarcinica-AJ131211 | 25 | 18 | 12 | 5 | 36 | 23 | 19 | 18 | 46 | 36 | 27 | 23 | 51 | 46 | 40 | 35 | 57 | 51 | 40 | 35 | 61 | 57 | 52 | 51 | 61 | 61 | 61 | 57 |
| N.farcinica-X91041 | 25 | 18 | 12 | 5 | 36 | 23 | 19 | 18 | 46 | 36 | 27 | 23 | 51 | 46 | 40 | 35 | 58 | 51 | 40 | 35 | 62 | 57 | 52 | 51 | 62 | 62 | 61 | 57 |
| N.farcinica-EF204470 | 25 | 18 | 12 | 5 | 36 | 23 | 19 | 18 | 47 | 36 | 27 | 23 | 52 | 47 | 40 | 35 | 59 | 52 | 40 | 35 | 63 | 58 | 53 | 52 | 63 | 63 | 62 | 58 |
| N.farcinica-X80595 | 25 | 18 | 12 | 5 | 36 | 23 | 19 | 18 | 48 | 36 | 27 | 23 | 53 | 48 | 40 | 35 | 59 | 53 | 40 | 35 | 63 | 59 | 54 | 53 | 63 | 63 | 63 | 59 |
| N.farcinica-X80604 | 25 | 18 | 12 | 5 | 36 | 23 | 19 | 18 | 48 | 36 | 27 | 23 | 53 | 48 | 40 | 35 | 59 | 53 | 40 | 35 | 63 | 59 | 54 | 53 | 63 | 63 | 63 | 59 |
| N.farcinica-Z36936 | 25 | 18 | 12 | 5 | 36 | 23 | 19 | 18 | 48 | 36 | 27 | 23 | 53 | 48 | 40 | 35 | 60 | 53 | 40 | 35 | 64 | 59 | 54 | 53 | 64 | 64 | 63 | 59 |
| N.farcinica-X80610 | 25 | 18 | 12 | 5 | 36 | 23 | 19 | 18 | 48 | 36 | 27 | 23 | 54 | 48 | 40 | 35 | 61 | 53 | 40 | 35 | 65 | 60 | 54 | 53 | 65 | 65 | 64 | 60 |
| N.farcinica-EF452728 | 25 | 18 | 12 | 5 | 36 | 23 | 19 | 18 | 48 | 36 | 27 | 23 | 54 | 48 | 40 | 35 | 61 | 53 | 40 | 35 | 65 | 60 | 54 | 53 | 65 | 65 | 64 | 60 |
| N.farcinica-AF430034 | 25 | 18 | 12 | 5 | 36 | 23 | 19 | 18 | 48 | 36 | 27 | 23 | 54 | 48 | 40 | 35 | 61 | 53 | 40 | 35 | 65 | 60 | 54 | 53 | 65 | 65 | 64 | 60 |
| N.farcinica-AB162792 | 25 | 18 | 12 | 5 | 36 | 23 | 19 | 18 | 48 | 36 | 27 | 23 | 54 | 48 | 40 | 35 | 62 | 53 | 40 | 35 | 66 | 61 | 54 | 53 | 66 | 66 | 65 | 61 |
| N.farcinica-AB162793 | 25 | 18 | 12 | 5 | 36 | 23 | 19 | 18 | 48 | 36 | 27 | 23 | 54 | 48 | 40 | 35 | 62 | 53 | 40 | 35 | 66 | 61 | 54 | 53 | 66 | 66 | 65 | 61 |
| N.farcinica-AY640108 | 25 | 18 | 12 | 5 | 36 | 23 | 19 | 18 | 48 | 36 | 27 | 23 | 54 | 48 | 40 | 35 | 62 | 53 | 40 | 35 | 67 | 61 | 54 | 53 | 67 | 67 | 66 | 61 |
| N.farcinica-AY640109 | 25 | 18 | 12 | 5 | 36 | 23 | 19 | 18 | 48 | 36 | 27 | 23 | 54 | 48 | 40 | 35 | 62 | 53 | 40 | 35 | 67 | 61 | 54 | 53 | 67 | 67 | 66 | 61 |
| N.farcinica-AY640110 | 25 | 18 | 12 | 5 | 36 | 23 | 19 | 18 | 48 | 36 | 27 | 23 | 54 | 48 | 40 | 35 | 62 | 53 | 40 | 35 | 67 | 61 | 54 | 53 | 67 | 67 | 66 | 61 |
| N.farcinica-AY640111 | 25 | 18 | 12 | 5 | 36 | 23 | 19 | 18 | 48 | 36 | 27 | 23 | 54 | 48 | 40 | 35 | 62 | 53 | 40 | 35 | 67 | 61 | 54 | 53 | 67 | 67 | 66 | 61 |
| Nfarcinica-AY640112 | 25 | 18 | 12 | 5 | 36 | 23 | 19 | 18 | 48 | 36 | 27 | 23 | 54 | 48 | 40 | 35 | 62 | 53 | 40 | 35 | 67 | 61 | 54 | 53 | 67 | 67 | 66 | 61 |
| N.otitidiscaviarum-X80611 | 25 | 18 | 12 | 5 | 36 | 23 | 19 | 18 | 48 | 36 | 27 | 23 | 54 | 48 | 40 | 35 | 62 | 53 | 40 | 35 | 67 | 61 | 54 | 53 | 67 | 67 | 66 | 61 |
| N.farcinica-AF430033 | 25 | 18 | 12 | 5 | 36 | 23 | 19 | 18 | 48 | 36 | 27 | 23 | 54 | 48 | 40 | 35 | 62 | 53 | 40 | 35 | 67 | 61 | 54 | 53 | 67 | 67 | 66 | 61 |
| N.farcinica-AB162791 | 25 | 18 | 12 | 5 | 36 | 23 | 19 | 18 | 48 | 36 | 27 | 23 | 54 | 48 | 40 | 35 | 63 | 53 | 40 | 35 | 68 | 62 | 55 | 53 | 68 | 68 | 67 | 62 |
| N.farcinica-AB162795 | 25 | 18 | 12 | 5 | 36 | 23 | 19 | 18 | 48 | 36 | 27 | 23 | 55 | 48 | 40 | 35 | 63 | 53 | 40 | 35 | 68 | 62 | 55 | 53 | 68 | 68 | 67 | 62 |
| N.farcinica-AB162794 | 25 | 18 | 12 | 5 | 37 | 23 | 19 | 18 | 49 | 37 | 27 | 23 | 56 | 49 | 41 | 36 | 64 | 54 | 41 | 36 | 69 | 63 | 56 | 54 | 69 | 69 | 68 | 63 |
| N.farcinica-DQ659906 | 25 | 18 | 12 | 5 | 37 | 23 | 19 | 18 | 49 | 37 | 27 | 23 | 56 | 49 | 41 | 36 | 64 | 54 | 41 | 36 | 69 | 63 | 56 | 54 | 69 | 69 | 68 | 63 |
| N.gamkensis-DQ235272 | 26 | 19 | 12 | 5 | 38 | 24 | 20 | 19 | 50 | 38 | 28 | 24 | 57 | 50 | 42 | 37 | 65 | 55 | 42 | 37 | 70 | 64 | 57 | 55 | 70 | 70 | 69 | 64 |
| Nexalbida-AB187522 | 26 | 19 | 12 | 5 | 39 | 24 | 20 | 19 | 51 | 39 | 28 | 24 | 57 | 51 | 43 | 37 | 66 | 56 | 43 | 37 | 71 | 65 | 58 | 56 | 71 | 71 | 70 | 65 |
| N.exalbida-AB187521 | 26 | 19 | 12 | 5 | 39 | 24 | 20 | 19 | 51 | 39 | 28 | 24 | 57 | 51 | 43 | 37 | 66 | 56 | 43 | 37 | 71 | 65 | 58 | 56 | 71 | 71 | 70 | 65 |
| N.beijingensis-AB094650 | 27 | 20 | 12 | 5 | 40 | 25 | 21 | 20 | 52 | 40 | 29 | 25 | 58 | 52 | 44 | 38 | 67 | 57 | 44 | 38 | 72 | 66 | 59 | 57 | 72 | 72 | 71 | 66 |
| N.asteroides-Z82228 | 27 | 20 | 12 | 5 | 40 | 25 | 21 | 20 | 52 | 40 | 30 | 25 | 59 | 52 | 44 | 39 | 67 | 57 | 44 | 39 | 72 | 66 | 59 | 57 | 72 | 72 | 71 | 66 |
| N.beijingensis-AB094647 | 27 | 20 | 12 | 5 | 40 | 25 | 21 | 20 | 52 | 40 | 30 | 25 | 59 | 52 | 44 | 39 | 67 | 57 | 44 | 39 | 72 | 66 | 59 | 57 | 72 | 72 | 71 | 66 |
| N.beijingensis-AF154129 | 27 | 20 | 12 | 5 | 40 | 25 | 21 | 20 | 52 | 40 | 30 | 25 | 59 | 52 | 44 | 39 | 67 | 57 | 44 | 39 | 73 | 66 | 59 | 57 | 73 | 73 | 72 | 66 |
| N.beijingensis-AB094640 | 27 | 20 | 12 | 5 | 40 | 25 | 21 | 20 | 52 | 40 | 30 | 25 | 59 | 52 | 44 | 39 | 67 | 57 | 44 | 39 | 73 | 66 | 59 | 57 | 73 | 73 | 72 | 66 |
| N.beijingensis-AB094646 | 27 | 20 | 12 | 5 | 40 | 25 | 21 | 20 | 53 | 40 | 30 | 25 | 60 | 52 | 44 | 39 | 68 | 58 | 44 | 39 | 74 | 67 | 60 | 58 | 74 | 74 | 73 | 67 |
| N.beijingensis-AB162628 | 27 | 20 | 12 | 5 | 41 | 25 | 21 | 20 | 54 | 41 | 30 | 25 | 61 | 53 | 45 | 40 | 69 | 59 | 45 | 40 | 75 | 68 | 61 | 59 | 75 | 75 | 74 | 68 |
| N.beijingensis-DQ659901 | 27 | 20 | 12 | 5 | 41 | 25 | 21 | 20 | 54 | 41 | 30 | 25 | 61 | 53 | 45 | 40 | 69 | 59 | 45 | 40 | 75 | 68 | 61 | 59 | 75 | 75 | 74 | 68 |
| N.beijingensis-AB094639 | 27 | 20 | 12 | 5 | 41 | 25 | 21 | 20 | 54 | 41 | 30 | 25 | 61 | 53 | 45 | 40 | 69 | 59 | 45 | 40 | 75 | 68 | 61 | 59 | 75 | 75 | 74 | 68 |
| N.beijingensis-AB094645 | 27 | 20 | 12 | 5 | 41 | 25 | 21 | 20 | 54 | 41 | 30 | 25 | 61 | 53 | 45 | 40 | 69 | 59 | 45 | 40 | 76 | 68 | 61 | 59 | 76 | 76 | 75 | 68 |
| N.beijingensis-AB094648 | 27 | 20 | 12 | 5 | 41 | 25 | 21 | 20 | 54 | 41 | 30 | 25 | 61 | 53 | 45 | 40 | 69 | 59 | 45 | 40 | 76 | 68 | 61 | 59 | 76 | 76 | 75 | 68 |
| N.beijingensis-AB094656 | 27 | 20 | 12 | 5 | 41 | 25 | 21 | 20 | 54 | 41 | 30 | 25 | 61 | 53 | 45 | 40 | 70 | 59 | 45 | 40 | 77 | 69 | 62 | 59 | 77 | 77 | 76 | 69 |
| N.beijingensis-AB094651 | 27 | 20 | 12 | 5 | 41 | 25 | 21 | 20 | 54 | 41 | 30 | 25 | 61 | 53 | 45 | 40 | 70 | 59 | 45 | 40 | 77 | 69 | 62 | 59 | 77 | 77 | 76 | 69 |
| N.beijingensis-AB094644 | 27 | 20 | 12 | 5 | 41 | 25 | 21 | 20 | 54 | 41 | 30 | 25 | 61 | 53 | 45 | 40 | 70 | 59 | 45 | 40 | 77 | 69 | 62 | 59 | 77 | 77 | 76 | 69 |
| N.beijingensis-AB094654 | 27 | 20 | 12 | 5 | 41 | 25 | 21 | 20 | 54 | 41 | 30 | 25 | 61 | 53 | 45 | 40 | 71 | 59 | 45 | 40 | 78 | 70 | 63 | 59 | 78 | 78 | 77 | 70 |
| N.beijingensis-AB162629 | 27 | 20 | 12 | 5 | 41 | 25 | 21 | 20 | 54 | 41 | 30 | 25 | 62 | 53 | 45 | 40 | 72 | 60 | 45 | 40 | 79 | 71 | 64 | 60 | 79 | 79 | 78 | 71 |
| N.beijingensis-AB094653 | 27 | 20 | 12 | 5 | 41 | 25 | 21 | 20 | 54 | 41 | 30 | 25 | 62 | 53 | 45 | 40 | 72 | 60 | 45 | 40 | 79 | 71 | 64 | 60 | 79 | 79 | 78 | 71 |
| N.beijingensis-AB094643 | 27 | 20 | 12 | 5 | 41 | 25 | 21 | 20 | 54 | 41 | 30 | 25 | 62 | 53 | 45 | 40 | 72 | 60 | 45 | 40 | 79 | 71 | 64 | 60 | 79 | 79 | 78 | 71 |
| N.beijingensis-AB094642 | 27 | 20 | 12 | 5 | 41 | 25 | 21 | 20 | 54 | 41 | 30 | 25 | 62 | 53 | 45 | 40 | 72 | 60 | 45 | 40 | 79 | 71 | 64 | 60 | 79 | 79 | 78 | 71 |
| N.beijingensis-AB094641 | 27 | 20 | 12 | 5 | 41 | 25 | 21 | 20 | 54 | 41 | 30 | 25 | 62 | 53 | 45 | 40 | 72 | 60 | 45 | 40 | 79 | 71 | 64 | 60 | 79 | 79 | 78 | 71 |
| N.beijingensis-AB094649 | 27 | 20 | 12 | 5 | 41 | 25 | 21 | 20 | 54 | 41 | 30 | 25 | 62 | 53 | 45 | 40 | 72 | 60 | 45 | 40 | 80 | 71 | 64 | 60 | 80 | 80 | 79 | 71 |
| N.beijingensis-AB094655 | 27 | 20 | 12 | 5 | 41 | 25 | 21 | 20 | 54 | 41 | 30 | 25 | 62 | 53 | 45 | 40 | 73 | 60 | 45 | 40 | 81 | 72 | 65 | 60 | 81 | 81 | 80 | 72 |
| N.beijingensis-AB094652 | 27 | 20 | 12 | 5 | 41 | 25 | 21 | 20 | 54 | 41 | 30 | 25 | 62 | 53 | 45 | 40 | 73 | 60 | 45 | 40 | 81 | 72 | 65 | 60 | 81 | 81 | 80 | 72 |
| N.araoensis-AB108779 | 27 | 20 | 12 | 5 | 42 | 25 | 21 | 20 | 55 | 42 | 30 | 25 | 63 | 54 | 46 | 41 | 74 | 61 | 46 | 41 | 82 | 73 | 65 | 61 | 82 | 82 | 81 | 73 |
| N.asteroides-Z82227 | 27 | 20 | 12 | 5 | 42 | 25 | 21 | 20 | 56 | 42 | 30 | 25 | 64 | 55 | 47 | 41 | 75 | 62 | 47 | 41 | 82 | 74 | 66 | 62 | 82 | 82 | 81 | 74 |
| N.arthritidis-AB212949 | 27 | 20 | 12 | 5 | 42 | 25 | 21 | 20 | 56 | 42 | 30 | 25 | 64 | 55 | 47 | 41 | 75 | 62 | 47 | 41 | 82 | 74 | 66 | 62 | 82 | 82 | 81 | 74 |
| N.arthritidis-AB108781 | 27 | 20 | 12 | 5 | 42 | 25 | 21 | 20 | 56 | 42 | 30 | 25 | 65 | 55 | 47 | 41 | 76 | 63 | 47 | 41 | 83 | 75 | 67 | 63 | 83 | 83 | 82 | 75 |
| N.arthritidis-DQ659896 | 27 | 20 | 12 | 5 | 42 | 25 | 21 | 20 | 56 | 42 | 30 | 25 | 65 | 55 | 47 | 41 | 76 | 63 | 47 | 41 | 83 | 75 | 67 | 63 | 83 | 83 | 82 | 75 |
| N.asiatica-AB092569 | 27 | 20 | 12 | 5 | 43 | 25 | 21 | 20 | 57 | 43 | 31 | 25 | 66 | 56 | 48 | 42 | 77 | 64 | 48 | 42 | 84 | 76 | 68 | 64 | 84 | 84 | 83 | 76 |
| N.asiatica-AB092567 | 27 | 20 | 12 | 5 | 43 | 25 | 21 | 20 | 57 | 43 | 31 | 25 | 66 | 56 | 48 | 42 | 77 | 64 | 48 | 42 | 84 | 76 | 68 | 64 | 84 | 84 | 83 | 76 |
| N.asiatica-AB162797 | 27 | 20 | 12 | 5 | 43 | 25 | 21 | 20 | 57 | 43 | 31 | 25 | 66 | 56 | 48 | 42 | 77 | 64 | 48 | 42 | 85 | 76 | 68 | 64 | 85 | 85 | 84 | 76 |
| N.asiatica-AB097456 | 27 | 20 | 12 | 5 | 43 | 25 | 21 | 20 | 57 | 43 | 31 | 25 | 66 | 56 | 48 | 42 | 77 | 64 | 48 | 42 | 85 | 76 | 68 | 64 | 85 | 85 | 84 | 76 |
| N.asiatica-AB092570 | 27 | 20 | 12 | 5 | 43 | 25 | 21 | 20 | 57 | 43 | 31 | 25 | 66 | 56 | 48 | 42 | 77 | 64 | 48 | 42 | 85 | 76 | 68 | 64 | 85 | 85 | 84 | 76 |
| N.asiatica-AB097457 | 27 | 20 | 12 | 5 | 43 | 25 | 21 | 20 | 57 | 43 | 31 | 25 | 66 | 56 | 48 | 42 | 78 | 64 | 48 | 42 | 86 | 77 | 69 | 64 | 86 | 86 | 85 | 77 |
| N.asiatica-AB162798 | 27 | 20 | 12 | 5 | 43 | 25 | 21 | 20 | 57 | 43 | 31 | 25 | 66 | 56 | 48 | 42 | 79 | 64 | 48 | 42 | 87 | 78 | 69 | 64 | 87 | 87 | 86 | 78 |
| N.asiatica-AB162796 | 27 | 20 | 12 | 5 | 43 | 25 | 21 | 20 | 57 | 43 | 31 | 25 | 66 | 56 | 48 | 42 | 79 | 64 | 48 | 42 | 87 | 78 | 69 | 64 | 87 | 87 | 86 | 78 |
| N.asiatica-AB097458 | 27 | 20 | 12 | 5 | 43 | 25 | 21 | 20 | 57 | 43 | 31 | 25 | 66 | 56 | 48 | 42 | 79 | 64 | 48 | 42 | 87 | 78 | 69 | 64 | 87 | 87 | 86 | 78 |
| N.asiatica-AB092568 | 27 | 20 | 12 | 5 | 43 | 25 | 21 | 20 | 58 | 43 | 31 | 25 | 67 | 57 | 48 | 42 | 80 | 65 | 48 | 42 | 88 | 79 | 70 | 65 | 88 | 88 | 87 | 79 |
| N.asiatica-AB092566 | 27 | 20 | 12 | 5 | 43 | 25 | 21 | 20 | 58 | 43 | 31 | 25 | 67 | 57 | 48 | 42 | 81 | 65 | 48 | 42 | 89 | 80 | 71 | 65 | 89 | 89 | 88 | 80 |
| N.asiatica-DQ659897 | 27 | 20 | 12 | 5 | 43 | 25 | 21 | 20 | 58 | 43 | 31 | 25 | 67 | 57 | 48 | 42 | 81 | 65 | 48 | 42 | 89 | 80 | 71 | 65 | 89 | 89 | 88 | 80 |
| N.abscessus-AF218293 | 27 | 20 | 12 | 5 | 44 | 25 | 21 | 20 | 59 | 44 | 32 | 25 | 68 | 58 | 49 | 43 | 82 | 66 | 49 | 43 | 90 | 81 | 72 | 66 | 90 | 90 | 89 | 81 |
| N.asteroides-X84851 | 27 | 20 | 12 | 5 | 44 | 25 | 21 | 20 | 59 | 44 | 32 | 25 | 68 | 58 | 49 | 43 | 82 | 66 | 49 | 43 | 90 | 81 | 72 | 66 | 90 | 90 | 89 | 81 |
| N.asteroides-Z82219 | 27 | 20 | 12 | 5 | 44 | 25 | 21 | 20 | 59 | 44 | 32 | 25 | 68 | 58 | 49 | 43 | 82 | 66 | 49 | 43 | 90 | 81 | 72 | 66 | 90 | 90 | 89 | 81 |
| N.abscessus-AB108774 | 27 | 20 | 12 | 5 | 44 | 25 | 21 | 20 | 59 | 44 | 32 | 25 | 69 | 58 | 49 | 43 | 83 | 67 | 49 | 43 | 91 | 82 | 73 | 67 | 91 | 91 | 90 | 82 |
| N.abscessus-AB162809 | 27 | 20 | 12 | 5 | 44 | 25 | 21 | 20 | 59 | 44 | 32 | 25 | 69 | 58 | 49 | 43 | 83 | 67 | 49 | 43 | 91 | 82 | 73 | 67 | 91 | 91 | 90 | 82 |
| N.abscessus-AB108771 | 27 | 20 | 12 | 5 | 44 | 25 | 21 | 20 | 60 | 44 | 32 | 25 | 70 | 59 | 50 | 43 | 84 | 68 | 50 | 43 | 92 | 83 | 74 | 68 | 92 | 92 | 91 | 83 |
| N.abscessus-AF430018 | 27 | 20 | 12 | 5 | 44 | 25 | 21 | 20 | 60 | 44 | 32 | 25 | 70 | 59 | 50 | 43 | 84 | 68 | 50 | 43 | 93 | 83 | 74 | 68 | 93 | 93 | 92 | 83 |
| N.abscessus-AF218292 | 27 | 20 | 12 | 5 | 44 | 25 | 21 | 20 | 60 | 44 | 32 | 25 | 70 | 59 | 50 | 43 | 84 | 68 | 50 | 43 | 93 | 83 | 74 | 68 | 93 | 93 | 92 | 83 |
| N.abscessus-DQ351151 | 27 | 20 | 12 | 5 | 44 | 25 | 21 | 20 | 60 | 44 | 32 | 25 | 70 | 59 | 50 | 43 | 84 | 68 | 50 | 43 | 93 | 83 | 74 | 68 | 93 | 93 | 92 | 83 |
| N.abscessus-DQ659895 | 27 | 20 | 12 | 5 | 44 | 25 | 21 | 20 | 60 | 44 | 32 | 25 | 70 | 59 | 50 | 43 | 84 | 68 | 50 | 43 | 93 | 83 | 74 | 68 | 93 | 93 | 92 | 83 |
| N.abscessus-AB115182 | 27 | 20 | 12 | 5 | 44 | 25 | 21 | 20 | 60 | 44 | 32 | 25 | 70 | 59 | 50 | 43 | 84 | 68 | 50 | 43 | 94 | 83 | 74 | 68 | 94 | 94 | 93 | 83 |
| N.abscessus-AB108773 | 27 | 20 | 12 | 5 | 44 | 25 | 21 | 20 | 60 | 44 | 32 | 25 | 70 | 59 | 50 | 43 | 84 | 68 | 50 | 43 | 94 | 83 | 74 | 68 | 94 | 94 | 93 | 83 |
| N.abscessus-AB162805 | 27 | 20 | 12 | 5 | 44 | 25 | 21 | 20 | 61 | 44 | 32 | 25 | 71 | 60 | 50 | 43 | 85 | 69 | 50 | 43 | 95 | 84 | 75 | 69 | 95 | 95 | 94 | 84 |
| N.abscessus-AB162808 | 27 | 20 | 12 | 5 | 44 | 25 | 21 | 20 | 61 | 44 | 32 | 25 | 71 | 60 | 50 | 43 | 85 | 69 | 50 | 43 | 95 | 84 | 75 | 69 | 95 | 95 | 94 | 84 |
| N.abscessus-AB162806 | 27 | 20 | 12 | 5 | 44 | 25 | 21 | 20 | 61 | 44 | 32 | 25 | 71 | 60 | 50 | 43 | 85 | 69 | 50 | 43 | 95 | 84 | 75 | 69 | 95 | 95 | 94 | 84 |
| N.abscessus-AB108770 | 27 | 20 | 12 | 5 | 44 | 25 | 21 | 20 | 61 | 44 | 32 | 25 | 71 | 60 | 50 | 43 | 85 | 69 | 50 | 43 | 96 | 84 | 75 | 69 | 96 | 96 | 95 | 84 |
| N.abscessus-AB162807 | 27 | 20 | 12 | 5 | 44 | 25 | 21 | 20 | 61 | 44 | 32 | 25 | 71 | 60 | 50 | 43 | 85 | 69 | 50 | 43 | 97 | 84 | 75 | 69 | 97 | 97 | 96 | 84 |
| N.abscessus-AB212947 | 27 | 20 | 12 | 5 | 44 | 25 | 21 | 20 | 61 | 44 | 32 | 25 | 71 | 60 | 50 | 43 | 85 | 69 | 50 | 43 | 97 | 84 | 75 | 69 | 97 | 97 | 96 | 84 |
| N.otitidiscaviarum-Z82234 | 28 | 21 | 13 | 5 | 45 | 26 | 22 | 21 | 62 | 45 | 33 | 26 | 72 | 61 | 51 | 44 | 86 | 70 | 51 | 44 | 98 | 85 | 76 | 70 | 98 | 98 | 97 | 85 |
| N.otitidiscaviarum-Z82238 | 28 | 21 | 13 | 5 | 45 | 26 | 22 | 21 | 62 | 45 | 33 | 26 | 72 | 61 | 51 | 44 | 86 | 70 | 51 | 44 | 98 | 85 | 76 | 70 | 98 | 98 | 97 | 85 |
| N.seriolae-EF513204 | 29 | 22 | 14 | 6 | 46 | 27 | 23 | 22 | 63 | 46 | 34 | 27 | 73 | 62 | 52 | 45 | 87 | 71 | 52 | 45 | 99 | 86 | 77 | 71 | 99 | 99 | 98 | 86 |
| N.crassostreae-AF430049 | 30 | 23 | 15 | 7 | 47 | 28 | 24 | 23 | 64 | 47 | 35 | 28 | 74 | 63 | 53 | 46 | 88 | 72 | 53 | 46 | 99 | 87 | 78 | 72 | 99 | 99 | 98 | 87 |
| N.crassostreae-U92799 | 30 | 23 | 15 | 7 | 47 | 28 | 24 | 23 | 64 | 47 | 35 | 28 | 74 | 63 | 53 | 46 | 88 | 72 | 53 | 46 | 99 | 87 | 78 | 72 | 99 | 99 | 98 | 87 |
| N.crassostreae-Z37989 | 30 | 23 | 15 | 7 | 47 | 28 | 24 | 23 | 64 | 47 | 35 | 28 | 74 | 63 | 53 | 46 | 88 | 72 | 53 | 46 | 100 | 87 | 78 | 72 | 100 | 100 | 99 | 87 |
| N.seriolae-AF380936 | 31 | 24 | 15 | 7 | 48 | 29 | 25 | 24 | 65 | 48 | 36 | 29 | 75 | 64 | 54 | 47 | 89 | 73 | 54 | 47 | 101 | 88 | 79 | 73 | 101 | 101 | 100 | 88 |
| N.seriolae-X80592 | 31 | 24 | 15 | 7 | 49 | 29 | 25 | 24 | 66 | 49 | 36 | 29 | 76 | 65 | 55 | 48 | 90 | 74 | 55 | 48 | 101 | 89 | 80 | 74 | 101 | 101 | 100 | 89 |
| N.seriolae-AY846841 | 31 | 24 | 15 | 7 | 49 | 29 | 25 | 24 | 66 | 49 | 36 | 29 | 76 | 65 | 55 | 48 | 90 | 74 | 55 | 48 | 102 | 89 | 80 | 74 | 102 | 102 | 101 | 89 |
| N.seriolae-AF254420 | 31 | 24 | 15 | 7 | 49 | 29 | 25 | 24 | 66 | 49 | 36 | 29 | 76 | 65 | 55 | 48 | 90 | 74 | 55 | 48 | 102 | 89 | 80 | 74 | 102 | 102 | 101 | 89 |
| N.seriolae-AB255702 | 31 | 24 | 15 | 7 | 49 | 29 | 25 | 24 | 66 | 49 | 36 | 29 | 76 | 65 | 55 | 48 | 90 | 74 | 55 | 48 | 102 | 89 | 80 | 74 | 102 | 102 | 101 | 89 |
| N.seriolae-AF254421 | 31 | 24 | 15 | 7 | 49 | 29 | 25 | 24 | 66 | 49 | 36 | 29 | 76 | 65 | 55 | 48 | 90 | 74 | 55 | 48 | 102 | 89 | 80 | 74 | 102 | 102 | 101 | 89 |
| N.seriolae-AF380937 | 31 | 24 | 15 | 7 | 49 | 29 | 25 | 24 | 66 | 49 | 36 | 29 | 76 | 65 | 55 | 48 | 90 | 74 | 55 | 48 | 102 | 89 | 80 | 74 | 102 | 102 | 101 | 89 |
| N.seriolae-EF192033 | 31 | 24 | 15 | 7 | 49 | 29 | 25 | 24 | 66 | 49 | 36 | 29 | 77 | 65 | 55 | 48 | 91 | 75 | 55 | 48 | 103 | 90 | 81 | 75 | 103 | 103 | 102 | 90 |
| N.seriolae-AF380938 | 31 | 24 | 15 | 7 | 49 | 29 | 25 | 24 | 66 | 49 | 36 | 29 | 77 | 65 | 55 | 48 | 91 | 75 | 55 | 48 | 103 | 90 | 81 | 75 | 103 | 103 | 102 | 90 |
| N.seriolae-AY017474 | 31 | 24 | 15 | 7 | 49 | 29 | 25 | 24 | 66 | 49 | 36 | 29 | 77 | 65 | 55 | 48 | 92 | 75 | 55 | 48 | 104 | 91 | 82 | 75 | 104 | 104 | 103 | 91 |
| N.seriolae-AF430039 | 31 | 24 | 15 | 7 | 49 | 29 | 25 | 24 | 66 | 49 | 36 | 29 | 77 | 65 | 55 | 48 | 92 | 75 | 55 | 48 | 104 | 91 | 82 | 75 | 104 | 104 | 103 | 91 |
| N.seriolae-Z36925 | 31 | 24 | 15 | 7 | 49 | 29 | 25 | 24 | 66 | 49 | 36 | 29 | 77 | 65 | 55 | 48 | 92 | 75 | 55 | 48 | 104 | 91 | 82 | 75 | 104 | 104 | 103 | 91 |
| N.seriolae-DQ659915 | 31 | 24 | 15 | 7 | 49 | 29 | 25 | 24 | 66 | 49 | 36 | 29 | 77 | 65 | 55 | 48 | 92 | 75 | 55 | 48 | 104 | 91 | 82 | 75 | 104 | 104 | 103 | 91 |
| N.seriolae-AB255699 | 31 | 24 | 15 | 7 | 49 | 29 | 25 | 24 | 66 | 49 | 36 | 29 | 77 | 65 | 55 | 48 | 92 | 75 | 55 | 48 | 104 | 91 | 82 | 75 | 104 | 104 | 103 | 91 |
| N.seriolae-AB255700 | 31 | 24 | 15 | 7 | 49 | 29 | 25 | 24 | 66 | 49 | 36 | 29 | 77 | 65 | 55 | 48 | 92 | 75 | 55 | 48 | 104 | 91 | 82 | 75 | 104 | 104 | 103 | 91 |
| N.seriolae-AB255701 | 31 | 24 | 15 | 7 | 49 | 29 | 25 | 24 | 66 | 49 | 36 | 29 | 77 | 65 | 55 | 48 | 92 | 75 | 55 | 48 | 104 | 91 | 82 | 75 | 104 | 104 | 103 | 91 |
| N.seriolae-AF251566 | 31 | 24 | 15 | 7 | 49 | 29 | 25 | 24 | 66 | 49 | 36 | 29 | 77 | 65 | 55 | 48 | 92 | 75 | 55 | 48 | 104 | 91 | 82 | 75 | 104 | 104 | 103 | 91 |
| N.seriolae-AF254418 | 31 | 24 | 15 | 7 | 49 | 29 | 25 | 24 | 66 | 49 | 36 | 29 | 77 | 65 | 55 | 48 | 92 | 75 | 55 | 48 | 104 | 91 | 82 | 75 | 104 | 104 | 103 | 91 |
| N.concava-EF177464 | 32 | 24 | 15 | 7 | 50 | 30 | 26 | 24 | 67 | 50 | 37 | 30 | 78 | 66 | 56 | 49 | 93 | 76 | 56 | 49 | 105 | 92 | 83 | 76 | 105 | 105 | 104 | 92 |
| N.concava-AB126880 | 32 | 24 | 15 | 7 | 51 | 30 | 26 | 24 | 68 | 50 | 37 | 30 | 79 | 66 | 56 | 49 | 94 | 77 | 56 | 49 | 106 | 93 | 84 | 76 | 106 | 106 | 105 | 93 |
| N.concava-AB126881 | 32 | 24 | 15 | 7 | 51 | 30 | 26 | 24 | 68 | 50 | 37 | 30 | 79 | 66 | 56 | 49 | 94 | 77 | 56 | 49 | 106 | 93 | 84 | 76 | 106 | 106 | 105 | 93 |
| N.areane-DQ282122 | 33 | 25 | 16 | 8 | 52 | 31 | 27 | 25 | 69 | 51 | 38 | 31 | 80 | 67 | 57 | 50 | 95 | 78 | 57 | 50 | 107 | 94 | 85 | 77 | 107 | 107 | 106 | 94 |
| N.harenae-DQ282122 | 33 | 25 | 16 | 8 | 52 | 31 | 27 | 25 | 69 | 51 | 38 | 31 | 80 | 67 | 57 | 50 | 95 | 78 | 57 | 50 | 107 | 94 | 85 | 77 | 107 | 107 | 106 | 94 |
| N.transvalensis-AB084447 | 34 | 26 | 16 | 8 | 53 | 32 | 28 | 26 | 70 | 52 | 39 | 32 | 81 | 68 | 58 | 51 | 96 | 79 | 58 | 51 | 108 | 95 | 86 | 78 | 108 | 108 | 107 | 95 |
| N.transvalensis-AB084448 | 34 | 26 | 16 | 8 | 53 | 32 | 28 | 26 | 70 | 52 | 39 | 32 | 81 | 68 | 58 | 51 | 96 | 79 | 58 | 51 | 108 | 95 | 86 | 78 | 108 | 108 | 107 | 95 |
| N.asteroides-Z82220 | 34 | 26 | 16 | 8 | 54 | 32 | 28 | 26 | 71 | 53 | 39 | 32 | 82 | 69 | 59 | 52 | 97 | 80 | 59 | 52 | 109 | 96 | 87 | 79 | 109 | 109 | 108 | 96 |
| N.asteroides-Z82221 | 34 | 26 | 16 | 8 | 54 | 32 | 28 | 26 | 71 | 53 | 39 | 32 | 82 | 69 | 59 | 52 | 97 | 80 | 59 | 52 | 109 | 96 | 87 | 79 | 109 | 109 | 108 | 96 |
| N.asteroides-Z82229 | 34 | 26 | 16 | 8 | 54 | 32 | 28 | 26 | 71 | 53 | 39 | 32 | 82 | 69 | 59 | 52 | 97 | 80 | 59 | 52 | 109 | 96 | 87 | 79 | 109 | 109 | 108 | 96 |
| N.asteroides-AY191251 | 34 | 26 | 16 | 8 | 54 | 32 | 28 | 26 | 71 | 53 | 39 | 32 | 82 | 69 | 59 | 52 | 98 | 80 | 59 | 52 | 110 | 97 | 87 | 79 | 110 | 110 | 109 | 97 |
| N.asteroides-DQ659899 | 34 | 26 | 16 | 8 | 54 | 32 | 28 | 26 | 71 | 53 | 39 | 32 | 82 | 69 | 59 | 52 | 98 | 80 | 59 | 52 | 110 | 97 | 87 | 79 | 110 | 110 | 109 | 97 |
| N.transvalensis-AB084445 | 34 | 26 | 16 | 8 | 54 | 32 | 28 | 26 | 71 | 53 | 39 | 32 | 82 | 69 | 59 | 52 | 98 | 80 | 59 | 52 | 111 | 97 | 87 | 79 | 111 | 111 | 110 | 97 |
| N.transvalensis-AB084446 | 34 | 26 | 16 | 8 | 54 | 32 | 28 | 26 | 72 | 53 | 39 | 32 | 83 | 70 | 59 | 52 | 99 | 81 | 59 | 52 | 112 | 98 | 88 | 80 | 112 | 112 | 111 | 98 |
| N.transvalensis-AB084444 | 34 | 26 | 16 | 8 | 54 | 32 | 28 | 26 | 72 | 53 | 39 | 32 | 83 | 70 | 59 | 52 | 99 | 81 | 59 | 52 | 112 | 98 | 88 | 80 | 112 | 112 | 111 | 98 |
| N.wallacei-EU099357 | 34 | 26 | 16 | 8 | 54 | 32 | 28 | 26 | 72 | 53 | 39 | 32 | 83 | 70 | 59 | 52 | 99 | 81 | 59 | 52 | 112 | 98 | 88 | 80 | 112 | 112 | 111 | 98 |
| N.transvalensis-X80598 | 35 | 26 | 16 | 8 | 55 | 33 | 28 | 26 | 73 | 54 | 40 | 33 | 84 | 71 | 60 | 53 | 100 | 82 | 60 | 53 | 113 | 99 | 89 | 81 | 113 | 113 | 112 | 99 |
| N.transvalensis-X80609 | 35 | 26 | 16 | 8 | 55 | 33 | 28 | 26 | 73 | 54 | 40 | 33 | 84 | 71 | 60 | 53 | 100 | 82 | 60 | 53 | 113 | 99 | 89 | 81 | 113 | 113 | 112 | 99 |
| N.transvalensis-AF430047 | 35 | 26 | 16 | 8 | 55 | 33 | 28 | 26 | 73 | 54 | 40 | 33 | 84 | 71 | 60 | 53 | 100 | 82 | 60 | 53 | 114 | 99 | 89 | 81 | 114 | 114 | 113 | 99 |
| N.transvalensis-DQ659916 | 35 | 26 | 16 | 8 | 55 | 33 | 28 | 26 | 73 | 54 | 40 | 33 | 84 | 71 | 60 | 53 | 100 | 82 | 60 | 53 | 114 | 99 | 89 | 81 | 114 | 114 | 113 | 99 |
| N.transvalensis-Z36926 | 35 | 26 | 16 | 8 | 55 | 33 | 28 | 26 | 73 | 54 | 40 | 33 | 84 | 71 | 60 | 53 | 100 | 82 | 60 | 53 | 114 | 99 | 89 | 81 | 114 | 114 | 113 | 99 |
| N.transvalensis-Z82232 | 35 | 26 | 16 | 8 | 55 | 33 | 28 | 26 | 74 | 54 | 40 | 33 | 85 | 72 | 60 | 53 | 101 | 83 | 60 | 53 | 115 | 100 | 90 | 82 | 115 | 115 | 114 | 100 |
| N.transvalensis-Z82240 | 35 | 26 | 16 | 8 | 55 | 33 | 28 | 26 | 74 | 54 | 40 | 33 | 85 | 72 | 60 | 53 | 101 | 83 | 60 | 53 | 115 | 100 | 90 | 82 | 115 | 115 | 114 | 100 |
| N.transvalensis-Z82235 | 35 | 26 | 16 | 8 | 56 | 33 | 28 | 26 | 75 | 55 | 41 | 33 | 86 | 73 | 61 | 54 | 102 | 84 | 61 | 54 | 116 | 101 | 91 | 83 | 116 | 116 | 115 | 101 |
| N.transvalensis-Z82236 | 35 | 26 | 16 | 8 | 56 | 33 | 28 | 26 | 75 | 55 | 41 | 33 | 86 | 73 | 61 | 54 | 102 | 84 | 61 | 54 | 116 | 101 | 91 | 83 | 116 | 116 | 115 | 101 |
| N.blacklockiae-EU099360 | 35 | 26 | 16 | 8 | 57 | 33 | 28 | 26 | 76 | 56 | 41 | 33 | 87 | 74 | 62 | 55 | 103 | 85 | 62 | 55 | 117 | 102 | 92 | 84 | 117 | 117 | 116 | 102 |
| N.transvalensis-Z82233 | 35 | 26 | 16 | 8 | 57 | 33 | 28 | 26 | 76 | 56 | 41 | 33 | 87 | 74 | 62 | 55 | 103 | 85 | 62 | 55 | 117 | 102 | 92 | 84 | 117 | 117 | 116 | 102 |
| N.vermiculata-AB126873 | 36 | 27 | 16 | 8 | 58 | 34 | 29 | 27 | 77 | 57 | 42 | 34 | 88 | 75 | 63 | 56 | 104 | 86 | 63 | 56 | 118 | 103 | 93 | 85 | 118 | 118 | 117 | 103 |
| N.vaccinii-X80597 | 37 | 27 | 16 | 8 | 59 | 35 | 29 | 27 | 78 | 58 | 43 | 35 | 89 | 76 | 64 | 57 | 105 | 87 | 64 | 57 | 119 | 104 | 94 | 86 | 119 | 119 | 118 | 104 |
| N.vaccinii-AF430045 | 37 | 27 | 16 | 8 | 59 | 35 | 29 | 27 | 78 | 58 | 43 | 35 | 89 | 76 | 64 | 57 | 105 | 87 | 64 | 57 | 119 | 104 | 94 | 86 | 119 | 119 | 118 | 104 |
| N.vaccinii-Z36927 | 37 | 27 | 16 | 8 | 59 | 35 | 29 | 27 | 78 | 58 | 43 | 35 | 89 | 76 | 64 | 57 | 105 | 87 | 64 | 57 | 119 | 104 | 94 | 86 | 119 | 119 | 118 | 104 |
| N.vaccinii-AY191252 | 37 | 27 | 16 | 8 | 59 | 35 | 29 | 27 | 78 | 58 | 43 | 35 | 89 | 76 | 64 | 57 | 105 | 87 | 64 | 57 | 119 | 104 | 94 | 86 | 119 | 119 | 118 | 104 |
| N.vaccinii-DQ659917 | 37 | 27 | 16 | 8 | 59 | 35 | 29 | 27 | 78 | 58 | 43 | 35 | 89 | 76 | 64 | 57 | 105 | 87 | 64 | 57 | 119 | 104 | 94 | 86 | 119 | 119 | 118 | 104 |
| N.cerradoensis-AF060790 | 38 | 27 | 16 | 8 | 60 | 36 | 30 | 27 | 79 | 59 | 44 | 36 | 90 | 77 | 65 | 58 | 106 | 88 | 65 | 58 | 120 | 105 | 95 | 87 | 120 | 120 | 119 | 105 |
| N.aobensis-AB126878 | 39 | 27 | 16 | 8 | 61 | 37 | 30 | 27 | 80 | 60 | 45 | 37 | 91 | 78 | 66 | 59 | 107 | 89 | 66 | 59 | 121 | 106 | 96 | 88 | 121 | 121 | 120 | 106 |
| N.aobensis-AB126879 | 39 | 27 | 16 | 8 | 61 | 37 | 30 | 27 | 80 | 60 | 45 | 37 | 91 | 78 | 66 | 59 | 107 | 89 | 66 | 59 | 121 | 106 | 96 | 88 | 121 | 121 | 120 | 106 |
| N.aobensis-AB126875 | 39 | 27 | 16 | 8 | 61 | 37 | 30 | 27 | 80 | 60 | 45 | 37 | 92 | 78 | 66 | 59 | 108 | 90 | 66 | 59 | 122 | 107 | 97 | 89 | 122 | 122 | 121 | 107 |
| N.aobensis-AB126876 | 39 | 27 | 16 | 8 | 61 | 37 | 30 | 27 | 81 | 60 | 45 | 37 | 92 | 79 | 66 | 59 | 108 | 90 | 66 | 59 | 122 | 107 | 97 | 89 | 122 | 122 | 121 | 107 |
| N.aobensis-AB126877 | 39 | 27 | 16 | 8 | 61 | 37 | 30 | 27 | 81 | 60 | 45 | 37 | 92 | 79 | 66 | 59 | 108 | 90 | 66 | 59 | 122 | 107 | 97 | 89 | 122 | 122 | 121 | 107 |
| N.veterana-AF430059 | 39 | 27 | 16 | 8 | 62 | 37 | 30 | 27 | 82 | 61 | 46 | 37 | 93 | 80 | 67 | 60 | 109 | 91 | 67 | 60 | 123 | 108 | 98 | 90 | 123 | 123 | 122 | 108 |
| N.kruczakiae-AY441974 | 39 | 27 | 16 | 8 | 63 | 37 | 30 | 27 | 82 | 62 | 46 | 37 | 94 | 80 | 68 | 61 | 110 | 92 | 68 | 61 | 124 | 109 | 99 | 91 | 124 | 124 | 123 | 109 |
| N.kruczakiae-DQ659909 | 39 | 27 | 16 | 8 | 63 | 37 | 30 | 27 | 82 | 62 | 46 | 37 | 94 | 80 | 68 | 61 | 110 | 92 | 68 | 61 | 124 | 109 | 99 | 91 | 124 | 124 | 123 | 109 |
| N.veterana-AF278572 | 39 | 27 | 16 | 8 | 63 | 37 | 30 | 27 | 82 | 62 | 46 | 37 | 95 | 80 | 68 | 61 | 111 | 93 | 68 | 61 | 125 | 110 | 100 | 92 | 125 | 125 | 124 | 110 |
| N.veterana-AF430055 | 39 | 27 | 16 | 8 | 63 | 37 | 30 | 27 | 82 | 62 | 46 | 37 | 95 | 80 | 68 | 61 | 111 | 93 | 68 | 61 | 125 | 110 | 100 | 92 | 125 | 125 | 124 | 110 |
| N.veterana-DQ659918 | 39 | 27 | 16 | 8 | 63 | 37 | 30 | 27 | 82 | 62 | 46 | 37 | 95 | 80 | 68 | 61 | 111 | 93 | 68 | 61 | 126 | 110 | 100 | 92 | 126 | 126 | 125 | 110 |
| N.veterana-AY191253 | 39 | 27 | 16 | 8 | 63 | 37 | 30 | 27 | 82 | 62 | 46 | 37 | 95 | 80 | 68 | 61 | 111 | 93 | 68 | 61 | 126 | 110 | 100 | 92 | 126 | 126 | 125 | 110 |
| N.veterana-AY171039 | 39 | 27 | 16 | 8 | 63 | 37 | 30 | 27 | 82 | 62 | 46 | 37 | 95 | 80 | 68 | 61 | 111 | 93 | 68 | 61 | 126 | 110 | 100 | 92 | 126 | 126 | 125 | 110 |
| N.veterana-AF490540 | 39 | 27 | 16 | 8 | 63 | 37 | 30 | 27 | 82 | 62 | 46 | 37 | 95 | 80 | 68 | 61 | 111 | 93 | 68 | 61 | 126 | 110 | 100 | 92 | 126 | 126 | 125 | 110 |
| N.veterana-AY149599 | 39 | 27 | 16 | 8 | 63 | 37 | 30 | 27 | 82 | 62 | 46 | 37 | 95 | 80 | 68 | 61 | 111 | 93 | 68 | 61 | 126 | 110 | 100 | 92 | 126 | 126 | 125 | 110 |
| N.elegans-AJ854057 | 39 | 27 | 16 | 8 | 64 | 37 | 30 | 27 | 83 | 63 | 47 | 37 | 96 | 81 | 69 | 62 | 112 | 94 | 69 | 62 | 127 | 111 | 101 | 93 | 127 | 127 | 126 | 111 |
| N.elegans-DQ659905 | 39 | 27 | 16 | 8 | 64 | 37 | 30 | 27 | 83 | 63 | 47 | 37 | 96 | 81 | 69 | 62 | 112 | 94 | 69 | 62 | 127 | 111 | 101 | 93 | 127 | 127 | 126 | 111 |
| N.elegans-AJ854058 | 39 | 27 | 16 | 8 | 64 | 37 | 30 | 27 | 83 | 63 | 47 | 37 | 96 | 81 | 69 | 62 | 112 | 94 | 69 | 62 | 127 | 111 | 101 | 93 | 127 | 127 | 126 | 111 |
| N.elegans-AB237142 | 39 | 27 | 16 | 8 | 64 | 37 | 30 | 27 | 83 | 63 | 47 | 37 | 96 | 81 | 69 | 62 | 112 | 94 | 69 | 62 | 127 | 111 | 101 | 93 | 127 | 127 | 126 | 111 |
| N.africana-AF277198 | 39 | 27 | 16 | 8 | 64 | 37 | 30 | 27 | 84 | 63 | 47 | 37 | 97 | 82 | 70 | 62 | 113 | 95 | 70 | 62 | 128 | 112 | 102 | 94 | 128 | 128 | 127 | 112 |
| N.africana-AF430054 | 39 | 27 | 16 | 8 | 64 | 37 | 30 | 27 | 84 | 63 | 47 | 37 | 97 | 82 | 70 | 62 | 114 | 95 | 70 | 62 | 129 | 113 | 103 | 94 | 129 | 129 | 128 | 113 |
| N.africana-AF302232 | 39 | 27 | 16 | 8 | 64 | 37 | 30 | 27 | 84 | 63 | 47 | 37 | 97 | 82 | 70 | 62 | 114 | 95 | 70 | 62 | 129 | 113 | 103 | 94 | 129 | 129 | 128 | 113 |
| N.africana-AY089701 | 39 | 27 | 16 | 8 | 64 | 37 | 30 | 27 | 84 | 63 | 47 | 37 | 98 | 82 | 70 | 62 | 114 | 96 | 70 | 62 | 129 | 113 | 103 | 95 | 129 | 129 | 128 | 113 |
| N.yamanashiensis-DQ659920 | 40 | 28 | 16 | 8 | 65 | 38 | 31 | 28 | 85 | 64 | 48 | 38 | 99 | 83 | 71 | 63 | 115 | 97 | 71 | 63 | 130 | 114 | 104 | 96 | 130 | 130 | 129 | 114 |
| N.yamanashiensi-AB092561 | 40 | 28 | 16 | 8 | 65 | 38 | 31 | 28 | 85 | 64 | 48 | 38 | 99 | 83 | 71 | 63 | 115 | 97 | 71 | 63 | 130 | 114 | 104 | 96 | 130 | 130 | 129 | 114 |
| N.inohanensis-AJ619769 | 40 | 28 | 16 | 8 | 66 | 38 | 31 | 28 | 86 | 65 | 48 | 38 | 100 | 84 | 72 | 64 | 116 | 98 | 72 | 64 | 131 | 115 | 105 | 97 | 131 | 131 | 130 | 115 |
| N.inohanensis-AB092560 | 40 | 28 | 16 | 8 | 66 | 38 | 31 | 28 | 87 | 65 | 48 | 38 | 100 | 85 | 73 | 64 | 117 | 98 | 73 | 64 | 132 | 116 | 106 | 97 | 132 | 132 | 131 | 116 |
| N.inohanensis-DQ659908 | 40 | 28 | 16 | 8 | 66 | 38 | 31 | 28 | 87 | 65 | 48 | 38 | 100 | 85 | 73 | 64 | 117 | 98 | 73 | 64 | 132 | 116 | 106 | 97 | 132 | 132 | 131 | 116 |
| N.uniformis-AF430044 | 41 | 28 | 16 | 8 | 67 | 39 | 32 | 28 | 88 | 66 | 49 | 39 | 101 | 86 | 74 | 65 | 118 | 99 | 74 | 65 | 133 | 117 | 107 | 98 | 133 | 133 | 132 | 117 |
| N.uniformis-Z46752 | 41 | 28 | 16 | 8 | 67 | 39 | 32 | 28 | 88 | 66 | 49 | 39 | 101 | 86 | 74 | 65 | 118 | 99 | 74 | 65 | 133 | 117 | 107 | 98 | 133 | 133 | 132 | 117 |
| N.niigatensis-AB092562 | 42 | 29 | 16 | 8 | 68 | 40 | 33 | 29 | 89 | 67 | 50 | 40 | 102 | 87 | 75 | 66 | 119 | 100 | 75 | 66 | 134 | 118 | 108 | 99 | 134 | 134 | 133 | 118 |
| N.niigatensis-AB092563 | 42 | 29 | 16 | 8 | 68 | 40 | 33 | 29 | 89 | 67 | 50 | 40 | 102 | 87 | 75 | 66 | 119 | 100 | 75 | 66 | 134 | 118 | 108 | 99 | 134 | 134 | 133 | 118 |
| N.niigatensis-AB092564 | 42 | 29 | 16 | 8 | 68 | 40 | 33 | 29 | 89 | 67 | 50 | 40 | 102 | 87 | 75 | 66 | 119 | 100 | 75 | 66 | 134 | 118 | 108 | 99 | 134 | 134 | 133 | 118 |
| N.niigatensis-AB092565 | 42 | 29 | 16 | 8 | 68 | 40 | 33 | 29 | 89 | 67 | 50 | 40 | 102 | 87 | 75 | 66 | 119 | 100 | 75 | 66 | 134 | 118 | 108 | 99 | 134 | 134 | 133 | 118 |
| N.niigatensis-DQ659910 | 42 | 29 | 16 | 8 | 68 | 40 | 33 | 29 | 89 | 67 | 50 | 40 | 102 | 87 | 75 | 66 | 119 | 100 | 75 | 66 | 134 | 118 | 108 | 99 | 134 | 134 | 133 | 118 |
| N.transvalensis-AB201301 | 43 | 30 | 16 | 8 | 69 | 41 | 34 | 30 | 90 | 68 | 51 | 41 | 103 | 88 | 76 | 67 | 120 | 101 | 76 | 67 | 135 | 119 | 109 | 100 | 135 | 135 | 134 | 119 |
| N.transvalensis-AB201302 | 44 | 30 | 16 | 8 | 70 | 41 | 34 | 30 | 91 | 69 | 51 | 41 | 104 | 89 | 77 | 68 | 121 | 102 | 77 | 68 | 136 | 120 | 110 | 101 | 136 | 136 | 135 | 120 |
| N.transvalensis-AB201300 | 44 | 30 | 16 | 8 | 70 | 41 | 34 | 30 | 91 | 69 | 51 | 41 | 104 | 89 | 77 | 68 | 121 | 102 | 77 | 68 | 136 | 120 | 110 | 101 | 136 | 136 | 135 | 120 |
| N.brasiliensis-AB201298 | 44 | 30 | 16 | 8 | 70 | 41 | 34 | 30 | 92 | 69 | 51 | 41 | 105 | 90 | 78 | 68 | 122 | 103 | 78 | 68 | 137 | 121 | 111 | 102 | 137 | 137 | 136 | 121 |
| N.brasiliensis-AB201299 | 44 | 30 | 16 | 8 | 70 | 41 | 34 | 30 | 92 | 69 | 51 | 41 | 105 | 90 | 78 | 68 | 122 | 103 | 78 | 68 | 137 | 121 | 111 | 102 | 137 | 137 | 136 | 121 |
| N.otitidiscaviarum-AB201303 | 44 | 30 | 16 | 8 | 70 | 41 | 34 | 30 | 92 | 69 | 51 | 41 | 105 | 90 | 78 | 68 | 122 | 103 | 78 | 68 | 137 | 121 | 111 | 102 | 137 | 137 | 136 | 121 |
| N.otitidiscaviarum-M59056 | 45 | 31 | 16 | 8 | 71 | 42 | 35 | 31 | 93 | 70 | 52 | 42 | 106 | 91 | 79 | 69 | 123 | 104 | 79 | 69 | 138 | 122 | 112 | 103 | 138 | 138 | 137 | 122 |
| N.otitidiscaviarum-AB110907 | 45 | 31 | 16 | 8 | 72 | 42 | 35 | 31 | 94 | 71 | 53 | 42 | 107 | 92 | 80 | 70 | 124 | 105 | 80 | 70 | 139 | 123 | 113 | 104 | 139 | 139 | 138 | 123 |
| N.otitidiscaviarum-X80599 | 45 | 31 | 16 | 8 | 72 | 42 | 35 | 31 | 94 | 71 | 53 | 42 | 107 | 92 | 80 | 70 | 124 | 105 | 80 | 70 | 139 | 123 | 113 | 104 | 139 | 139 | 138 | 123 |
| N.otitidiscaviarum-EU031786 | 45 | 31 | 16 | 8 | 72 | 42 | 35 | 31 | 94 | 71 | 53 | 42 | 107 | 92 | 80 | 70 | 124 | 105 | 80 | 70 | 139 | 123 | 113 | 104 | 139 | 139 | 138 | 123 |
| N.otitidiscaviarum-AF430067 | 45 | 31 | 16 | 8 | 72 | 42 | 35 | 31 | 94 | 71 | 53 | 42 | 107 | 92 | 80 | 70 | 124 | 105 | 80 | 70 | 139 | 123 | 113 | 104 | 139 | 139 | 138 | 123 |
| N.otitidiscaviarum-DQ659912 | 45 | 31 | 16 | 8 | 72 | 42 | 35 | 31 | 94 | 71 | 53 | 42 | 107 | 92 | 80 | 70 | 124 | 105 | 80 | 70 | 139 | 123 | 113 | 104 | 139 | 139 | 138 | 123 |
| N.otitidiscaviarum-AF430068 | 45 | 31 | 16 | 8 | 72 | 42 | 35 | 31 | 94 | 71 | 53 | 42 | 107 | 92 | 80 | 70 | 124 | 105 | 80 | 70 | 140 | 123 | 113 | 104 | 140 | 140 | 139 | 123 |
| N.otitidiscaviarum-EU203569 | 45 | 31 | 16 | 8 | 72 | 42 | 35 | 31 | 94 | 71 | 53 | 42 | 107 | 92 | 80 | 70 | 124 | 105 | 80 | 70 | 140 | 123 | 113 | 104 | 140 | 140 | 139 | 123 |
| N.otitidiscaviarum-EU203570 | 45 | 31 | 16 | 8 | 72 | 42 | 35 | 31 | 94 | 71 | 53 | 42 | 107 | 92 | 80 | 70 | 124 | 105 | 80 | 70 | 140 | 123 | 113 | 104 | 140 | 140 | 139 | 123 |
| N.miyunensis-AY639901 | 46 | 32 | 16 | 8 | 73 | 43 | 36 | 32 | 95 | 72 | 54 | 43 | 108 | 93 | 81 | 71 | 125 | 106 | 81 | 71 | 141 | 124 | 114 | 105 | 141 | 141 | 140 | 124 |
| N.jiangxiensis-AY639902 | 46 | 32 | 16 | 8 | 73 | 43 | 36 | 32 | 96 | 72 | 54 | 43 | 109 | 93 | 81 | 71 | 126 | 107 | 81 | 71 | 142 | 125 | 115 | 106 | 142 | 142 | 141 | 125 |
| N.nova-DQ840030 | 46 | 32 | 16 | 8 | 73 | 43 | 36 | 32 | 96 | 72 | 54 | 43 | 109 | 93 | 81 | 71 | 126 | 107 | 81 | 71 | 142 | 125 | 115 | 106 | 142 | 142 | 141 | 125 |
| N.nova-AB162788 | 46 | 32 | 16 | 8 | 74 | 43 | 36 | 32 | 97 | 73 | 54 | 43 | 110 | 94 | 82 | 72 | 127 | 108 | 82 | 72 | 143 | 126 | 116 | 107 | 143 | 143 | 142 | 126 |
| N.nova-X80593 | 46 | 32 | 16 | 8 | 74 | 43 | 36 | 32 | 97 | 73 | 54 | 43 | 110 | 95 | 82 | 72 | 127 | 108 | 82 | 72 | 143 | 126 | 116 | 107 | 143 | 143 | 142 | 126 |
| N.nova-AB162789 | 46 | 32 | 16 | 8 | 74 | 43 | 36 | 32 | 97 | 73 | 54 | 43 | 110 | 95 | 82 | 72 | 128 | 108 | 82 | 72 | 144 | 127 | 116 | 107 | 144 | 144 | 143 | 127 |
| N.nova-AB162785 | 46 | 32 | 16 | 8 | 74 | 43 | 36 | 32 | 97 | 73 | 54 | 43 | 110 | 95 | 82 | 72 | 128 | 108 | 82 | 72 | 144 | 127 | 116 | 107 | 144 | 144 | 143 | 127 |
| N.nova-AF430030 | 46 | 32 | 16 | 8 | 74 | 43 | 36 | 32 | 97 | 73 | 54 | 43 | 110 | 95 | 82 | 72 | 128 | 108 | 82 | 72 | 144 | 127 | 116 | 107 | 144 | 144 | 143 | 127 |
| N.nova-DQ840026 | 46 | 32 | 16 | 8 | 74 | 43 | 36 | 32 | 97 | 73 | 54 | 43 | 110 | 95 | 82 | 72 | 128 | 108 | 82 | 72 | 144 | 127 | 116 | 107 | 144 | 144 | 143 | 127 |
| N.nova-AB162783 | 46 | 32 | 16 | 8 | 74 | 43 | 36 | 32 | 98 | 73 | 54 | 43 | 111 | 96 | 83 | 72 | 129 | 109 | 83 | 72 | 145 | 128 | 117 | 108 | 145 | 145 | 144 | 128 |
| N.nova-AF430031 | 46 | 32 | 16 | 8 | 74 | 43 | 36 | 32 | 99 | 73 | 54 | 43 | 112 | 97 | 83 | 72 | 130 | 110 | 83 | 72 | 146 | 129 | 118 | 109 | 146 | 146 | 145 | 129 |
| N.nova-AF430029 | 46 | 32 | 16 | 8 | 74 | 43 | 36 | 32 | 99 | 73 | 54 | 43 | 112 | 97 | 83 | 72 | 130 | 110 | 83 | 72 | 146 | 129 | 118 | 109 | 146 | 146 | 145 | 129 |
| N.nova-AB162790 | 46 | 32 | 16 | 8 | 74 | 43 | 36 | 32 | 99 | 73 | 54 | 43 | 113 | 97 | 83 | 72 | 131 | 110 | 83 | 72 | 147 | 130 | 118 | 109 | 147 | 147 | 146 | 130 |
| N.nova-AF430028 | 46 | 32 | 16 | 8 | 74 | 43 | 36 | 32 | 99 | 73 | 54 | 43 | 113 | 97 | 83 | 72 | 131 | 110 | 83 | 72 | 147 | 130 | 118 | 109 | 147 | 147 | 146 | 130 |
| N.nova-Z36930 | 46 | 32 | 16 | 8 | 74 | 43 | 36 | 32 | 99 | 73 | 54 | 43 | 113 | 97 | 83 | 72 | 131 | 110 | 83 | 72 | 147 | 130 | 118 | 109 | 147 | 147 | 146 | 130 |
| N.nova-AB162787 | 46 | 32 | 16 | 8 | 74 | 43 | 36 | 32 | 99 | 73 | 54 | 43 | 114 | 97 | 83 | 72 | 132 | 111 | 83 | 72 | 148 | 131 | 119 | 110 | 148 | 148 | 147 | 131 |
| N.nova-DQ659911 | 46 | 32 | 16 | 8 | 74 | 43 | 36 | 32 | 99 | 73 | 54 | 43 | 114 | 97 | 83 | 72 | 132 | 111 | 83 | 72 | 149 | 131 | 119 | 110 | 149 | 149 | 148 | 131 |
| N.nova-AY191250 | 46 | 32 | 16 | 8 | 74 | 43 | 36 | 32 | 99 | 73 | 54 | 43 | 114 | 97 | 83 | 72 | 132 | 111 | 83 | 72 | 149 | 131 | 119 | 110 | 149 | 149 | 148 | 131 |
| N.nova-AB162786 | 46 | 32 | 16 | 8 | 74 | 43 | 36 | 32 | 100 | 73 | 54 | 43 | 115 | 98 | 83 | 72 | 133 | 112 | 83 | 72 | 149 | 132 | 120 | 111 | 149 | 149 | 148 | 132 |
| N.nova-AF430032 | 46 | 32 | 16 | 8 | 74 | 43 | 36 | 32 | 101 | 73 | 54 | 43 | 116 | 99 | 84 | 72 | 134 | 113 | 84 | 72 | 150 | 133 | 121 | 112 | 150 | 150 | 149 | 133 |
| N.nova-AB162784 | 46 | 32 | 16 | 8 | 74 | 43 | 36 | 32 | 101 | 73 | 54 | 43 | 116 | 99 | 84 | 72 | 134 | 113 | 84 | 72 | 150 | 133 | 121 | 112 | 150 | 150 | 149 | 133 |
| N.jiangxiensis-DQ840027 | 47 | 32 | 16 | 8 | 75 | 44 | 36 | 32 | 102 | 74 | 55 | 44 | 117 | 100 | 85 | 73 | 135 | 114 | 85 | 73 | 151 | 134 | 122 | 113 | 151 | 151 | 150 | 134 |
| N.nova-DQ840028 | 47 | 32 | 16 | 8 | 75 | 44 | 36 | 32 | 102 | 74 | 55 | 44 | 117 | 100 | 85 | 73 | 135 | 114 | 85 | 73 | 152 | 134 | 122 | 113 | 152 | 152 | 151 | 134 |
| N.nova-DQ840029 | 47 | 32 | 16 | 8 | 75 | 44 | 36 | 32 | 102 | 74 | 55 | 44 | 117 | 100 | 85 | 73 | 135 | 114 | 85 | 73 | 152 | 134 | 122 | 113 | 152 | 152 | 151 | 134 |
| N.anaemiae-AB162801 | 48 | 32 | 16 | 8 | 76 | 45 | 36 | 32 | 103 | 75 | 56 | 45 | 118 | 101 | 86 | 74 | 136 | 115 | 86 | 74 | 153 | 135 | 123 | 114 | 153 | 153 | 152 | 135 |
| N.pseudovaccinii-AF430046 | 49 | 32 | 16 | 8 | 77 | 45 | 36 | 32 | 104 | 75 | 56 | 45 | 119 | 102 | 86 | 74 | 137 | 116 | 86 | 74 | 153 | 136 | 124 | 115 | 153 | 153 | 152 | 136 |
| N.vinacea-AB162802 | 49 | 32 | 16 | 8 | 77 | 45 | 36 | 32 | 104 | 76 | 57 | 45 | 119 | 102 | 87 | 75 | 137 | 116 | 87 | 75 | 154 | 136 | 124 | 115 | 154 | 154 | 153 | 136 |
| N.vinacea-AB024312 | 49 | 32 | 16 | 8 | 77 | 45 | 36 | 32 | 104 | 76 | 57 | 45 | 120 | 102 | 87 | 75 | 138 | 117 | 87 | 75 | 154 | 137 | 125 | 116 | 154 | 154 | 153 | 137 |
| N.vinacea-DQ659919 | 49 | 32 | 16 | 8 | 77 | 45 | 36 | 32 | 104 | 76 | 57 | 45 | 120 | 102 | 87 | 75 | 138 | 117 | 87 | 75 | 154 | 137 | 125 | 116 | 154 | 154 | 153 | 137 |
| N.acidivorans-AM402972 | 50 | 33 | 16 | 8 | 78 | 46 | 37 | 33 | 105 | 77 | 58 | 46 | 121 | 103 | 88 | 76 | 139 | 118 | 88 | 76 | 155 | 138 | 126 | 117 | 155 | 155 | 154 | 138 |
| N.pseudobrasiliensis-AB086861 | 51 | 33 | 16 | 8 | 79 | 47 | 37 | 33 | 106 | 78 | 59 | 47 | 121 | 104 | 89 | 77 | 139 | 118 | 89 | 77 | 156 | 138 | 126 | 117 | 156 | 156 | 155 | 138 |
| N.pseudobrasiliensis-AB086862 | 51 | 33 | 16 | 8 | 79 | 47 | 37 | 33 | 106 | 78 | 59 | 47 | 122 | 104 | 89 | 77 | 140 | 119 | 89 | 77 | 156 | 139 | 127 | 118 | 156 | 156 | 155 | 139 |
| N.pseudobrasiliensis-AB080196 | 51 | 33 | 16 | 8 | 80 | 47 | 37 | 33 | 107 | 79 | 59 | 47 | 122 | 105 | 90 | 77 | 140 | 119 | 90 | 77 | 157 | 139 | 127 | 118 | 157 | 157 | 156 | 139 |
| N.pseudobrasiliensis-AF430043 | 51 | 33 | 16 | 8 | 80 | 47 | 37 | 33 | 107 | 79 | 59 | 47 | 123 | 105 | 90 | 77 | 141 | 120 | 90 | 77 | 157 | 140 | 128 | 119 | 157 | 157 | 156 | 140 |
| N.pseudobrasiliensis-AF430042 | 51 | 33 | 16 | 8 | 80 | 47 | 37 | 33 | 107 | 79 | 59 | 47 | 123 | 105 | 90 | 77 | 141 | 120 | 90 | 77 | 158 | 140 | 128 | 119 | 158 | 158 | 157 | 140 |
| N.pseudobrasiliensis-DQ659914 | 51 | 33 | 16 | 8 | 80 | 47 | 37 | 33 | 107 | 79 | 59 | 47 | 123 | 105 | 90 | 77 | 141 | 120 | 90 | 77 | 158 | 140 | 128 | 119 | 158 | 158 | 157 | 140 |
| N.pseudobrasiliensis-X84853 | 51 | 33 | 16 | 8 | 80 | 47 | 37 | 33 | 107 | 79 | 59 | 47 | 123 | 105 | 90 | 77 | 141 | 120 | 90 | 77 | 158 | 140 | 128 | 119 | 158 | 158 | 157 | 140 |
| N.pseudobrasiliensis-X84855 | 51 | 33 | 16 | 8 | 80 | 47 | 37 | 33 | 107 | 79 | 59 | 47 | 123 | 105 | 90 | 77 | 141 | 120 | 90 | 77 | 159 | 140 | 128 | 119 | 159 | 159 | 158 | 140 |
| N.pseudobrasiliensis-X84854 | 51 | 33 | 16 | 8 | 80 | 47 | 37 | 33 | 108 | 79 | 59 | 47 | 124 | 106 | 91 | 77 | 142 | 121 | 91 | 77 | 160 | 141 | 129 | 120 | 160 | 160 | 159 | 141 |
| N.pseudobrasiliensis-X84852 | 51 | 33 | 16 | 8 | 80 | 47 | 37 | 33 | 108 | 79 | 59 | 47 | 124 | 106 | 91 | 77 | 142 | 121 | 91 | 77 | 160 | 141 | 129 | 120 | 160 | 160 | 159 | 141 |
