# Supplementary material for: Defining Reference Sequences for Nocardia Species by Similarity and Clustering Analyses of 16S rRNA Gene Sequence Data
Source: PLoS One. 2011 Jun 8;6(6):e19517. doi: 10.1371/journal.pone.0019517 (PMC3110597; doi:10.1371/journal.pone.0019517)
Supplement: Table S3 — Nocardia clustering results for k-means, hierarchical clustering algorithms, Cluto software, and PCA scores, clustered on the Euclidean distance for the first two principal components using the linear mapping clustering algorithm. (DOC) [file pone.0019517.s005.doc]

**Table S3.** Nocardia clustering results for k-means, hierarchical clustering (HC) algorithms, Cluto software, and PCA scores. Every two columns are sorted according to the clusters generated by the method used

| **Strain** | **KM** | **Strain** | **HC** | **Strain** | **Cluto** | **Strain** | **PCA** |
| --- | --- | --- | --- | --- | --- | --- | --- |
| asteroides-X57949 | 1 | transvalensis-X80598 | 1 | seriolae-AB060281 | 1 | seriolae-AB060281 | 1 |
| cyriacigeorgica-AB094570 | 1 | transvalensis-X80609 | 1 | seriolae-AB060282 | 1 | seriolae-AB060282 | 1 |
| cyriacigeorgica-AB094567 | 1 | transvalensis-AF430047 | 1 | seriolae-EF513204 | 2 | asteroides-X53205 | 2 |
| cyriacigeorgica-AB115949 | 1 | transvalensis-DQ659916 | 1 | soli-AF277223 | 3 | globerula-AF430065 | 3 |
| cyriacigeorgica-AB115952 | 1 | transvalensis-Z36926 | 1 | globerula-AF430065 | 4 | globerula-DQ525592 | 3 |
| cyriacigeorgica-AF282889 | 1 | transvalensis-Z82232 | 1 | globerula-DQ525592 | 4 | corynebacterioides-AF430066 | 4 |
| cyriacigeorgica-AF430020 | 1 | transvalensis-Z82240 | 2 | jejuensis-AY964666 | 5 | corynebacterioides-AY438619 | 4 |
| cyriacigeorgica-AF430027 | 1 | transvalensis-Z82235 | 2 | pigrifrangens-AF219974 | 6 | soli-AF277223 | 5 |
| cyriacigeorgica-AB115951 | 1 | transvalensis-Z82236 | 2 | corynebacterioides-AF430066 | 7 | coubleae-DQ235688 | 5 |
| cyriacigeorgica-AB094585 | 1 | blacklockiae-EU099360 | 2 | corynebacterioides-AY438619 | 7 | ignorata-AJ303008 | 5 |
| coubleae-DQ235688 | 2 | transvalensis-Z82233 | 2 | seriolae-AF380936 | 8 | ignorata-AY191254 | 5 |
| jejuensis-AY964666 | 2 | asteroides-Z82231 | 3 | seriolae-X80592 | 8 | ignorata-DQ659907 | 5 |
| tenerifensis-AJ556157 | 3 | jinanensis-DQ462650 | 4 | seriolae-AY846841 | 8 | soli-AF277199 | 5 |
| iowensis-DQ925490 | 3 | speluncae-AM422449 | 4 | seriolae-AF254420 | 8 | soli-AF430051 | 5 |
| cyriacigeorgica-AB115948 | 4 | testacea-AB121769 | 5 | seriolae-AB255702 | 8 | cummidelens-AF277202 | 5 |
| cyriacigeorgica-AJ508414 | 4 | carnea-X80602 | 6 | seriolae-AF254421 | 8 | salmonicida-Z46750 | 6 |
| seriolae-AY846841 | 5 | carnea-AF430035 | 6 | seriolae-AF380937 | 8 | salmonicida-AF430050 | 6 |
| seriolae-AF254420 | 5 | carnea-X80607 | 6 | seriolae-EF192033 | 8 | fluminea-AF277204 | 6 |
| seriolae-AF254421 | 5 | carnea-Z36929 | 6 | seriolae-AF380938 | 8 | fluminea-AF430053 | 6 |
| seriolae-AF380937 | 5 | carnea-AF430036 | 6 | seriolae-AY017474 | 8 | pigrifrangens-AF219974 | 7 |
| seriolae-AY017474 | 5 | carnea-AF430037 | 6 | seriolae-AF430039 | 8 | jejuensis-AY964666 | 7 |
| seriolae-AF430039 | 5 | sienata-AB121770 | 6 | seriolae-Z36925 | 8 | alba-EU249584 | 8 |
| seriolae-AF251566 | 5 | testacea-AB192415 | 6 | seriolae-DQ659915 | 8 | ninae-DQ235687 | 8 |
| seriolae-AF254418 | 5 | flavorosea-AF430048 | 6 | seriolae-AB255699 | 8 | alba-AY222321 | 9 |
| asteroides-Z82228 | 6 | flavorosea-Z46754 | 6 | seriolae-AB255700 | 8 | mexicana-AY555577 | 10 |
| beijingensis-AF154129 | 6 | asteroides-AF163818 | 6 | seriolae-AB255701 | 8 | mexicana-AY560655 | 10 |
| beijingensis-AB094640 | 6 | asteroides-Z82230 | 6 | seriolae-AF251566 | 8 | caishijiensis-AF459443 | 11 |
| beijingensis-AB094646 | 6 | transvalensis-AB201302 | 7 | seriolae-AF254418 | 8 | carnea-X80602 | 11 |
| beijingensis-DQ659901 | 6 | miyunensis-AY639901 | 8 | otitidiscaviarum-AF475084 | 9 | carnea-AF430035 | 11 |
| beijingensis-AB094639 | 6 | asteroides-X57949 | 9 | coubleae-DQ235688 | 10 | carnea-X80607 | 11 |
| beijingensis-AB094645 | 6 | asteroides-Z82218 | 9 | ignorata-AJ303008 | 10 | carnea-Z36929 | 11 |
| beijingensis-AB094648 | 6 | cyriacigeorgica-DQ267485 | 9 | ignorata-AY191254 | 10 | carnea-AF430036 | 11 |
| beijingensis-AB094656 | 6 | cyriacigeorgica-AB094570 | 9 | ignorata-DQ659907 | 10 | carnea-AF430037 | 11 |
| beijingensis-AB094651 | 6 | cyriacigeorgica-EF127500 | 9 | arthritidis-AB212949 | 11 | testacea-AB121769 | 11 |
| beijingensis-AB094644 | 6 | cyriacigeorgica-AB094581 | 9 | asiatica-AB092569 | 12 | sienata-AB121770 | 11 |
| beijingensis-AB094654 | 6 | cyriacigeorgica-AB094579 | 9 | asiatica-AB092567 | 12 | testacea-AB192415 | 11 |
| beijingensis-AB162629 | 6 | cyriacigeorgica-AB094569 | 9 | asiatica-AB162797 | 12 | flavorosea-AF430048 | 11 |
| beijingensis-AB094653 | 6 | cyriacigeorgica-AB094568 | 9 | asiatica-AB097456 | 12 | flavorosea-Z46754 | 11 |
| beijingensis-AB094643 | 6 | cyriacigeorgica-AB094565 | 9 | asiatica-AB092570 | 12 | asteroides-AF163818 | 11 |
| beijingensis-AB094642 | 6 | cyriacigeorgica-AB094566 | 9 | asiatica-AB097457 | 12 | asteroides-Z82230 | 11 |
| beijingensis-AB094641 | 6 | cyriacigeorgica-AB094567 | 9 | asiatica-AB162798 | 12 | asteroides-Z82231 | 11 |
| beijingensis-AB094649 | 6 | cyriacigeorgica-AB094571 | 9 | asiatica-AB162796 | 12 | jinanensis-DQ462650 | 12 |
| beijingensis-AB094655 | 6 | cyriacigeorgica-AB094573 | 9 | asiatica-AB097458 | 12 | speluncae-AM422449 | 12 |
| beijingensis-AB094652 | 6 | cyriacigeorgica-AB094574 | 9 | asiatica-AB092566 | 12 | tenerifensis-AJ556157 | 13 |
| asteroides-Z82231 | 7 | cyriacigeorgica-AB094578 | 9 | asiatica-DQ659897 | 12 | altamirensis-EU006090 | 13 |
| jinanensis-DQ462650 | 7 | cyriacigeorgica-AB094580 | 9 | asiatica-AB092568 | 13 | iowensis-DQ925490 | 13 |
| speluncae-AM422449 | 7 | cyriacigeorgica-AB094582 | 9 | soli-AF277199 | 14 | brasiliensis-X80591 | 14 |
| seriolae-X80592 | 8 | cyriacigeorgica-AB115949 | 9 | soli-AF430051 | 14 | brasiliensis-AY245543 | 14 |
| seriolae-AB255702 | 8 | cyriacigeorgica-AB115950 | 9 | cummidelens-AF277202 | 14 | brasiliensis-Z36935 | 14 |
| seriolae-EF192033 | 8 | cyriacigeorgica-AB115952 | 9 | salmonicida-Z46750 | 14 | brasiliensis-AF430038 | 14 |
| seriolae-AF380938 | 8 | cyriacigeorgica-AB115953 | 9 | salmonicida-AF430050 | 14 | brasiliensis-X80608 | 14 |
| seriolae-Z36925 | 8 | cyriacigeorgica-AB115954 | 9 | fluminea-AF277204 | 14 | brasiliensis-DQ659902 | 14 |
| seriolae-DQ659915 | 8 | cyriacigeorgica-AB115955 | 9 | fluminea-AF430053 | 14 | brevicatena-Z36928 | 15 |
| seriolae-AB255699 | 8 | cyriacigeorgica-AF282889 | 9 | asteroides-X57949 | 15 | brevicatena-X80600 | 16 |
| seriolae-AB255700 | 8 | cyriacigeorgica-AF430020 | 9 | asteroides-Z82218 | 15 | brevicatena-AF430040 | 16 |
| seriolae-AB255701 | 8 | cyriacigeorgica-AF430027 | 9 | cyriacigeorgica-DQ267485 | 15 | brevicatena-DQ659903 | 16 |
| takedensis-AB158277 | 9 | cyriacigeorgica-AY244782 | 9 | cyriacigeorgica-AB094570 | 15 | paucivorans-AJ437308 | 16 |
| takedensis-AB158278 | 9 | cyriacigeorgica-DQ303128 | 9 | cyriacigeorgica-EF127500 | 15 | paucivorans-AF430041 | 16 |
| takedensis-DQ840025 | 9 | cyriacigeorgica-EF127498 | 9 | cyriacigeorgica-AB094581 | 15 | paucivorans-AF179865 | 16 |
| nova-AB162783 | 10 | cyriacigeorgica-EF127499 | 9 | cyriacigeorgica-AB094579 | 15 | paucivorans-DQ659913 | 16 |
| nova-AB162790 | 10 | cyriacigeorgica-EF127501 | 9 | cyriacigeorgica-AB094569 | 15 | puris-AB097453 | 17 |
| nova-AF430032 | 10 | cyriacigeorgica-EF127502 | 9 | cyriacigeorgica-AB094568 | 15 | puris-AB097454 | 17 |
| nova-AB162784 | 10 | asteroides-DQ659900 | 9 | cyriacigeorgica-AB094565 | 15 | puris-AJ508748 | 17 |
| concava-EF177464 | 11 | asteroides-AF162772 | 9 | cyriacigeorgica-AB094566 | 15 | puris-AB097455 | 17 |
| concava-AB126880 | 11 | cyriacigeorgica-AB115951 | 9 | cyriacigeorgica-AB094567 | 15 | takedensis-AB158277 | 18 |
| concava-AB126881 | 11 | cyriacigeorgica-AB094585 | 9 | cyriacigeorgica-AB094571 | 15 | takedensis-AB158278 | 18 |
| uniformis-AF430044 | 11 | cyriacigeorgica-AB094575 | 9 | cyriacigeorgica-AB094573 | 15 | takedensis-DQ840025 | 18 |
| uniformis-Z46752 | 11 | cyriacigeorgica-AB094584 | 9 | cyriacigeorgica-AB094574 | 15 | amamiensis-AB275164 | 19 |
| puris-AB097453 | 12 | cyriacigeorgica-DQ659904 | 9 | cyriacigeorgica-AB094578 | 15 | thailandica-AB126874 | 20 |
| puris-AB097454 | 12 | cyriacigeorgica-AB094583 | 9 | cyriacigeorgica-AB094580 | 15 | neocaledoniensis-AY282603 | 20 |
| puris-AJ508748 | 12 | cyriacigeorgica-AB115948 | 10 | cyriacigeorgica-AB094582 | 15 | asteroides-AF430025 | 21 |
| puris-AB097455 | 12 | cyriacigeorgica-AJ508414 | 10 | cyriacigeorgica-AB115949 | 15 | asteroides-AF430026 | 21 |
| seriolae-AB060281 | 13 | cyriacigeorgica-AB094577 | 10 | cyriacigeorgica-AB115950 | 15 | asteroides-X84850 | 21 |
| seriolae-AB060282 | 13 | cyriacigeorgica-AB094572 | 10 | cyriacigeorgica-AB115952 | 15 | asteroides-X80606 | 21 |
| pigrifrangens-AF219974 | 14 | aobensis-AB126878 | 11 | cyriacigeorgica-AB115953 | 15 | asteroides-Z36934 | 21 |
| higoensis-AB108778 | 15 | aobensis-AB126879 | 12 | cyriacigeorgica-AB115954 | 15 | asteroides-AF430019 | 21 |
| asiatica-AB092568 | 15 | aobensis-AB126875 | 12 | cyriacigeorgica-AB115955 | 15 | nova-AB292584 | 21 |
| neocaledoniensis-AY282603 | 16 | aobensis-AB126876 | 12 | cyriacigeorgica-AF282889 | 15 | asteroides-DQ659898 | 21 |
| aobensis-AB126879 | 17 | aobensis-AB126877 | 12 | cyriacigeorgica-AF430020 | 15 | cyriacigeorgica-AB094576 | 22 |
| aobensis-AB126875 | 17 | veterana-AF430059 | 12 | cyriacigeorgica-AF430027 | 15 | asteroides-X57949 | 23 |
| aobensis-AB126876 | 17 | kruczakiae-AY441974 | 12 | cyriacigeorgica-AY244782 | 15 | asteroides-Z82218 | 23 |
| aobensis-AB126877 | 17 | kruczakiae-DQ659909 | 12 | cyriacigeorgica-DQ303128 | 15 | cyriacigeorgica-DQ267485 | 23 |
| elegans-AJ854057 | 17 | veterana-AF278572 | 12 | cyriacigeorgica-EF127498 | 15 | cyriacigeorgica-AB094570 | 23 |
| elegans-DQ659905 | 17 | veterana-AF430055 | 12 | cyriacigeorgica-EF127499 | 15 | cyriacigeorgica-EF127500 | 23 |
| elegans-AJ854058 | 17 | veterana-DQ659918 | 12 | cyriacigeorgica-EF127501 | 15 | cyriacigeorgica-AB115948 | 24 |
| elegans-AB237142 | 17 | veterana-AY191253 | 12 | cyriacigeorgica-EF127502 | 15 | cyriacigeorgica-AB094581 | 25 |
| africana-AF430054 | 17 | veterana-AY171039 | 12 | asteroides-DQ659900 | 15 | cyriacigeorgica-AB094579 | 25 |
| africana-AF302232 | 17 | veterana-AF490540 | 12 | asteroides-AF162772 | 15 | cyriacigeorgica-AJ508414 | 25 |
| aobensis-AB126878 | 18 | veterana-AY149599 | 12 | cyriacigeorgica-AB115951 | 15 | cyriacigeorgica-AB094569 | 25 |
| veterana-AF430059 | 18 | africana-AF277198 | 12 | cyriacigeorgica-AB094585 | 15 | cyriacigeorgica-AB094568 | 25 |
| kruczakiae-AY441974 | 18 | africana-AF430054 | 12 | cyriacigeorgica-AB094575 | 15 | cyriacigeorgica-AB094565 | 25 |
| kruczakiae-DQ659909 | 18 | africana-AF302232 | 12 | cyriacigeorgica-AB094584 | 15 | cyriacigeorgica-AB094566 | 25 |
| veterana-AF278572 | 18 | africana-AY089701 | 12 | cyriacigeorgica-DQ659904 | 15 | cyriacigeorgica-AB094567 | 25 |
| veterana-AF430055 | 18 | nova-X80593 | 13 | cyriacigeorgica-AB094583 | 15 | cyriacigeorgica-AB094571 | 25 |
| veterana-DQ659918 | 18 | nova-AB162789 | 13 | asteroides-Z82228 | 16 | cyriacigeorgica-AB094573 | 25 |
| veterana-AY191253 | 18 | nova-AB162785 | 13 | beijingensis-AF154129 | 16 | cyriacigeorgica-AB094574 | 25 |
| veterana-AY171039 | 18 | nova-AF430030 | 13 | beijingensis-AB094640 | 16 | cyriacigeorgica-AB094578 | 25 |
| veterana-AF490540 | 18 | nova-DQ840026 | 13 | beijingensis-AB094646 | 16 | cyriacigeorgica-AB094580 | 25 |
| veterana-AY149599 | 18 | nova-AB162783 | 13 | beijingensis-DQ659901 | 16 | cyriacigeorgica-AB094582 | 25 |
| africana-AF277198 | 18 | nova-AF430031 | 13 | beijingensis-AB094639 | 16 | cyriacigeorgica-AB115949 | 25 |
| africana-AY089701 | 18 | nova-AF430029 | 13 | beijingensis-AB094645 | 16 | cyriacigeorgica-AB115950 | 25 |
| abscessus-AB162809 | 19 | nova-AB162790 | 13 | beijingensis-AB094648 | 16 | cyriacigeorgica-AB115952 | 25 |
| abscessus-AB162808 | 19 | nova-AF430028 | 13 | beijingensis-AB094656 | 16 | cyriacigeorgica-AB115953 | 25 |
| abscessus-AB162807 | 19 | nova-Z36930 | 13 | beijingensis-AB094651 | 16 | cyriacigeorgica-AB115954 | 25 |
| yamanashiensis-DQ659920 | 20 | nova-AB162787 | 13 | beijingensis-AB094644 | 16 | cyriacigeorgica-AB115955 | 25 |
| ignorata-AJ303008 | 21 | nova-DQ659911 | 13 | beijingensis-AB094654 | 16 | cyriacigeorgica-AF282889 | 25 |
| ignorata-AY191254 | 21 | nova-AY191250 | 13 | beijingensis-AB162629 | 16 | cyriacigeorgica-AF430020 | 25 |
| ignorata-DQ659907 | 21 | nova-AB162786 | 13 | beijingensis-AB094653 | 16 | cyriacigeorgica-AF430027 | 25 |
| soli-AF277199 | 21 | nova-AF430032 | 13 | beijingensis-AB094643 | 16 | cyriacigeorgica-AY244782 | 25 |
| soli-AF430051 | 21 | nova-AB162784 | 13 | beijingensis-AB094642 | 16 | cyriacigeorgica-DQ303128 | 25 |
| cummidelens-AF277202 | 21 | jiangxiensis-AY639902 | 14 | beijingensis-AB094641 | 16 | cyriacigeorgica-EF127498 | 25 |
| salmonicida-Z46750 | 21 | nova-DQ840030 | 14 | beijingensis-AB094649 | 16 | cyriacigeorgica-EF127499 | 25 |
| salmonicida-AF430050 | 21 | jiangxiensis-DQ840027 | 14 | beijingensis-AB094655 | 16 | cyriacigeorgica-EF127501 | 25 |
| fluminea-AF277204 | 21 | nova-DQ840028 | 14 | beijingensis-AB094652 | 16 | cyriacigeorgica-EF127502 | 25 |
| fluminea-AF430053 | 21 | nova-DQ840029 | 14 | pneumoniae-AB108780 | 17 | asteroides-DQ659900 | 25 |
| transvalensis-AB201302 | 22 | ignorata-AJ303008 | 15 | niigatensis-AB092562 | 18 | asteroides-AF162772 | 25 |
| miyunensis-AY639901 | 22 | ignorata-AY191254 | 15 | niigatensis-AB092563 | 18 | cyriacigeorgica-AB094577 | 25 |
| otitidiscaviarum-AF475084 | 23 | ignorata-DQ659907 | 15 | niigatensis-AB092564 | 18 | cyriacigeorgica-AB115951 | 25 |
| cyriacigeorgica-DQ267485 | 24 | soli-AF277199 | 16 | niigatensis-AB092565 | 18 | cyriacigeorgica-AB094585 | 25 |
| cyriacigeorgica-AB094565 | 24 | soli-AF430051 | 16 | niigatensis-DQ659910 | 18 | cyriacigeorgica-AB094575 | 25 |
| cyriacigeorgica-AB094582 | 24 | cummidelens-AF277202 | 16 | concava-EF177464 | 19 | cyriacigeorgica-AB094572 | 25 |
| cyriacigeorgica-AB115950 | 24 | salmonicida-Z46750 | 16 | concava-AB126880 | 19 | cyriacigeorgica-AB094584 | 26 |
| cyriacigeorgica-AB115954 | 24 | salmonicida-AF430050 | 16 | concava-AB126881 | 19 | cyriacigeorgica-DQ659904 | 26 |
| testacea-AB121769 | 25 | fluminea-AF277204 | 16 | farcinica-AJ131211 | 20 | cyriacigeorgica-AB094583 | 26 |
| sienata-AB121770 | 25 | fluminea-AF430053 | 16 | farcinica-X91041 | 20 | pneumoniae-AB108780 | 27 |
| testacea-AB192415 | 25 | elegans-AJ854057 | 17 | farcinica-X80595 | 20 | polyresistens-AY626158 | 28 |
| asteroides-AF163818 | 25 | elegans-DQ659905 | 17 | farcinica-X80604 | 20 | lijiangensis-AY779043 | 28 |
| asteroides-Z82230 | 25 | elegans-AJ854058 | 17 | farcinica-Z36936 | 20 | xishanensis-AY333115 | 28 |
| asteroides-X53205 | 26 | elegans-AB237142 | 17 | farcinica-X80610 | 20 | higoensis-AB108778 | 29 |
| seriolae-EF513204 | 27 | uniformis-AF430044 | 18 | farcinica-EF452728 | 20 | shimofusensis-AB108777 | 30 |
| crassostreae-AF430049 | 28 | uniformis-Z46752 | 18 | farcinica-AF430034 | 20 | shimofusensis-AB108775 | 30 |
| crassostreae-U92799 | 28 | concava-EF177464 | 19 | farcinica-AB162792 | 20 | shimofusensis-AB108776 | 30 |
| crassostreae-Z37989 | 28 | concava-AB126880 | 19 | farcinica-AB162793 | 20 | otitidiscaviarum-AF475084 | 31 |
| niigatensis-AB092562 | 28 | concava-AB126881 | 19 | farcinica-AY640108 | 20 | farcinica-AJ131211 | 31 |
| niigatensis-AB092563 | 28 | anaemiae-AB162801 | 20 | farcinica-AY640109 | 20 | farcinica-X91041 | 31 |
| niigatensis-AB092564 | 28 | vinacea-AB162802 | 20 | farcinica-AY640110 | 20 | farcinica-EF204470 | 31 |
| niigatensis-AB092565 | 28 | vinacea-AB024312 | 20 | farcinica-AY640111 | 20 | farcinica-X80595 | 31 |
| niigatensis-DQ659910 | 28 | vinacea-DQ659919 | 20 | farcinica-AY640112 | 20 | farcinica-X80604 | 31 |
| brevicatena-Z36928 | 29 | brevicatena-Z36928 | 21 | otitidiscaviarum-X80611 | 20 | farcinica-Z36936 | 31 |
| araoensis-AB108779 | 30 | brevicatena-X80600 | 22 | farcinica-AF430033 | 20 | farcinica-X80610 | 31 |
| asteroides-Z82227 | 30 | brevicatena-AF430040 | 22 | farcinica-AB162791 | 20 | farcinica-EF452728 | 31 |
| arthritidis-AB108781 | 30 | brevicatena-DQ659903 | 22 | farcinica-AB162795 | 20 | farcinica-AF430034 | 31 |
| arthritidis-DQ659896 | 30 | paucivorans-AJ437308 | 22 | farcinica-AB162794 | 20 | farcinica-AB162792 | 31 |
| cyriacigeorgica-AB094569 | 31 | paucivorans-AF430041 | 22 | farcinica-DQ659906 | 20 | farcinica-AB162793 | 31 |
| cyriacigeorgica-AB094568 | 31 | paucivorans-AF179865 | 22 | beijingensis-AB094650 | 21 | farcinica-AY640108 | 31 |
| cyriacigeorgica-AB094566 | 31 | paucivorans-DQ659913 | 22 | beijingensis-AB094647 | 21 | farcinica-AY640109 | 31 |
| cyriacigeorgica-AB094571 | 31 | tenerifensis-AJ556157 | 23 | beijingensis-AB162628 | 21 | farcinica-AY640110 | 31 |
| cyriacigeorgica-AB094573 | 31 | iowensis-DQ925490 | 23 | nova-X80593 | 22 | farcinica-AY640111 | 31 |
| cyriacigeorgica-AB094574 | 31 | altamirensis-EU006090 | 24 | nova-AB162789 | 22 | farcinica-AY640112 | 31 |
| cyriacigeorgica-AB094580 | 31 | brasiliensis-X80591 | 24 | nova-AB162785 | 22 | otitidiscaviarum-X80611 | 31 |
| cyriacigeorgica-AB115953 | 31 | brasiliensis-AY245543 | 24 | nova-AF430030 | 22 | farcinica-AF430033 | 31 |
| cyriacigeorgica-AY244782 | 31 | brasiliensis-Z36935 | 24 | nova-DQ840026 | 22 | farcinica-AB162791 | 31 |
| asteroides-DQ659900 | 31 | brasiliensis-AF430038 | 24 | nova-AB162783 | 22 | farcinica-AB162795 | 31 |
| asteroides-AF162772 | 31 | brasiliensis-X80608 | 24 | nova-AF430031 | 22 | farcinica-AB162794 | 31 |
| nova-X80593 | 32 | brasiliensis-DQ659902 | 24 | nova-AF430029 | 22 | farcinica-DQ659906 | 31 |
| nova-DQ659911 | 32 | transvalensis-AB201301 | 25 | nova-AB162790 | 22 | gamkensis-DQ235272 | 32 |
| nova-AY191250 | 32 | transvalensis-AB201300 | 26 | nova-AF430028 | 22 | exalbida-AB187522 | 32 |
| acidivorans-AM402972 | 33 | brasiliensis-AB201298 | 26 | nova-Z36930 | 22 | exalbida-AB187521 | 32 |
| pseudobrasiliensis-AB086862 | 33 | brasiliensis-AB201299 | 26 | nova-DQ659911 | 22 | beijingensis-AB094650 | 33 |
| nova-AB162786 | 34 | otitidiscaviarum-AB201303 | 26 | nova-AY191250 | 22 | asteroides-Z82228 | 34 |
| arthritidis-AB212949 | 35 | niigatensis-AB092562 | 27 | nova-AB162786 | 22 | beijingensis-AB094647 | 34 |
| alba-EU249584 | 36 | niigatensis-AB092563 | 27 | nova-AF430032 | 22 | beijingensis-AF154129 | 34 |
| ninae-DQ235687 | 36 | niigatensis-AB092564 | 27 | nova-AB162784 | 22 | beijingensis-AB094640 | 34 |
| asteroides-X84851 | 37 | niigatensis-AB092565 | 27 | uniformis-AF430044 | 23 | beijingensis-AB094646 | 34 |
| asteroides-Z82219 | 37 | niigatensis-DQ659910 | 27 | uniformis-Z46752 | 23 | beijingensis-AB162628 | 34 |
| abscessus-DQ351151 | 37 | alba-AY222321 | 28 | pseudovaccinii-AF430046 | 24 | beijingensis-DQ659901 | 34 |
| abscessus-DQ659895 | 37 | caishijiensis-AF459443 | 29 | nova-AB162788 | 25 | beijingensis-AB094639 | 34 |
| abscessus-AB115182 | 37 | polyresistens-AY626158 | 30 | nova-AB162787 | 25 | beijingensis-AB094645 | 34 |
| abscessus-AB162806 | 37 | mexicana-AY555577 | 31 | transvalensis-AB201301 | 26 | beijingensis-AB094648 | 34 |
| abscessus-AB108770 | 37 | mexicana-AY560655 | 31 | abscessus-AF218293 | 27 | beijingensis-AB094656 | 34 |
| abscessus-AB212947 | 37 | amamiensis-AB275164 | 32 | asteroides-X84851 | 27 | beijingensis-AB094651 | 34 |
| brasiliensis-X80591 | 38 | otitidiscaviarum-Z82234 | 33 | asteroides-Z82219 | 27 | beijingensis-AB094644 | 34 |
| brasiliensis-AY245543 | 38 | otitidiscaviarum-Z82238 | 33 | abscessus-AB108774 | 27 | beijingensis-AB094654 | 34 |
| brasiliensis-Z36935 | 38 | asiatica-AB092569 | 34 | abscessus-AB162809 | 27 | beijingensis-AB162629 | 34 |
| brasiliensis-AF430038 | 38 | asiatica-AB092567 | 34 | abscessus-AB108771 | 27 | beijingensis-AB094653 | 34 |
| brasiliensis-X80608 | 38 | asiatica-AB162797 | 34 | abscessus-AF430018 | 27 | beijingensis-AB094643 | 34 |
| brasiliensis-DQ659902 | 38 | asiatica-AB097456 | 34 | abscessus-AF218292 | 27 | beijingensis-AB094642 | 34 |
| cyriacigeorgica-AB094581 | 39 | asiatica-AB092570 | 34 | abscessus-DQ351151 | 27 | beijingensis-AB094641 | 34 |
| cyriacigeorgica-AB094575 | 39 | asiatica-AB097457 | 34 | abscessus-DQ659895 | 27 | beijingensis-AB094649 | 34 |
| cyriacigeorgica-DQ659904 | 39 | asiatica-AB162798 | 34 | abscessus-AB115182 | 27 | beijingensis-AB094655 | 34 |
| cyriacigeorgica-AB094583 | 39 | asiatica-AB162796 | 34 | abscessus-AB108773 | 27 | beijingensis-AB094652 | 34 |
| thailandica-AB126874 | 40 | asiatica-AB097458 | 34 | abscessus-AB162805 | 27 | araoensis-AB108779 | 34 |
| abscessus-AF218293 | 41 | asiatica-AB092566 | 34 | abscessus-AB162808 | 27 | asteroides-Z82227 | 34 |
| abscessus-AB108771 | 41 | asiatica-DQ659897 | 34 | abscessus-AB162806 | 27 | arthritidis-AB212949 | 35 |
| abscessus-AF430018 | 41 | farcinica-AJ131211 | 35 | abscessus-AB108770 | 27 | arthritidis-AB108781 | 36 |
| abscessus-AF218292 | 41 | farcinica-X91041 | 35 | abscessus-AB162807 | 27 | arthritidis-DQ659896 | 36 |
| abscessus-AB108773 | 41 | farcinica-EF204470 | 35 | abscessus-AB212947 | 27 | asiatica-AB092569 | 36 |
| amamiensis-AB275164 | 42 | farcinica-X80595 | 35 | asteroides-X53205 | 28 | asiatica-AB092567 | 36 |
| pneumoniae-AB108780 | 42 | farcinica-X80604 | 35 | otitidiscaviarum-M59056 | 29 | asiatica-AB162797 | 36 |
| mexicana-AY555577 | 43 | farcinica-Z36936 | 35 | otitidiscaviarum-AB110907 | 29 | asiatica-AB097456 | 36 |
| mexicana-AY560655 | 43 | farcinica-X80610 | 35 | otitidiscaviarum-X80599 | 29 | asiatica-AB092570 | 36 |
| polyresistens-AY626158 | 43 | farcinica-EF452728 | 35 | otitidiscaviarum-EU031786 | 29 | asiatica-AB097457 | 36 |
| alba-AY222321 | 44 | farcinica-AF430034 | 35 | otitidiscaviarum-AF430067 | 29 | asiatica-AB162798 | 36 |
| caishijiensis-AF459443 | 44 | farcinica-AB162792 | 35 | otitidiscaviarum-DQ659912 | 29 | asiatica-AB162796 | 36 |
| asteroides-Z82218 | 45 | farcinica-AB162793 | 35 | otitidiscaviarum-AF430068 | 29 | asiatica-AB097458 | 36 |
| cyriacigeorgica-AB094579 | 45 | farcinica-AY640108 | 35 | otitidiscaviarum-EU203569 | 29 | asiatica-AB092568 | 37 |
| cyriacigeorgica-DQ303128 | 45 | farcinica-AY640109 | 35 | otitidiscaviarum-EU203570 | 29 | asiatica-AB092566 | 37 |
| cyriacigeorgica-AB094584 | 45 | farcinica-AY640110 | 35 | africana-AF277198 | 30 | asiatica-DQ659897 | 37 |
| asiatica-AB092569 | 46 | farcinica-AY640111 | 35 | africana-AF430054 | 30 | abscessus-AF218293 | 38 |
| asiatica-AB092567 | 46 | farcinica-AY640112 | 35 | africana-AF302232 | 30 | asteroides-X84851 | 38 |
| asiatica-AB092566 | 46 | otitidiscaviarum-X80611 | 35 | africana-AY089701 | 30 | asteroides-Z82219 | 38 |
| asiatica-DQ659897 | 46 | farcinica-AF430033 | 35 | higoensis-AB108778 | 31 | abscessus-AB108774 | 38 |
| areane-DQ282122 | 47 | farcinica-AB162791 | 35 | farcinica-EF204470 | 31 | abscessus-AB162809 | 38 |
| harenae-DQ282122 | 47 | farcinica-AB162795 | 35 | transvalensis-AB201300 | 32 | abscessus-AB108771 | 38 |
| yamanashiensi-AB092561 | 48 | farcinica-AB162794 | 35 | brasiliensis-AB201298 | 32 | abscessus-AF430018 | 38 |
| inohanensis-AJ619769 | 48 | farcinica-DQ659906 | 35 | brasiliensis-AB201299 | 32 | abscessus-AF218292 | 38 |
| inohanensis-AB092560 | 48 | otitidiscaviarum-AF475084 | 36 | cerradoensis-AF060790 | 33 | abscessus-DQ351151 | 38 |
| inohanensis-DQ659908 | 48 | lijiangensis-AY779043 | 37 | aobensis-AB126878 | 33 | abscessus-DQ659895 | 38 |
| anaemiae-AB162801 | 49 | xishanensis-AY333115 | 37 | veterana-AF430059 | 34 | abscessus-AB115182 | 38 |
| pseudovaccinii-AF430046 | 49 | gamkensis-DQ235272 | 38 | kruczakiae-AY441974 | 34 | abscessus-AB108773 | 38 |
| vinacea-AB162802 | 49 | exalbida-AB187522 | 38 | kruczakiae-DQ659909 | 34 | abscessus-AB162805 | 38 |
| vinacea-AB024312 | 49 | exalbida-AB187521 | 38 | veterana-AF278572 | 34 | abscessus-AB162808 | 38 |
| vinacea-DQ659919 | 49 | acidivorans-AM402972 | 39 | veterana-AF430055 | 34 | abscessus-AB162806 | 38 |
| cyriacigeorgica-AB094576 | 50 | crassostreae-AF430049 | 40 | veterana-DQ659918 | 34 | abscessus-AB108770 | 38 |
| cyriacigeorgica-AB094577 | 50 | crassostreae-U92799 | 40 | veterana-AY191253 | 34 | abscessus-AB162807 | 38 |
| cyriacigeorgica-AB094572 | 50 | crassostreae-Z37989 | 40 | veterana-AY171039 | 34 | abscessus-AB212947 | 38 |
| transvalensis-Z82240 | 51 | higoensis-AB108778 | 41 | veterana-AF490540 | 34 | otitidiscaviarum-Z82234 | 39 |
| transvalensis-Z82235 | 51 | puris-AB097453 | 42 | veterana-AY149599 | 34 | otitidiscaviarum-Z82238 | 39 |
| transvalensis-Z82236 | 51 | puris-AB097454 | 42 | pseudobrasiliensis-AB086862 | 35 | seriolae-EF513204 | 40 |
| blacklockiae-EU099360 | 51 | puris-AJ508748 | 42 | araoensis-AB108779 | 36 | crassostreae-AF430049 | 41 |
| transvalensis-Z82233 | 51 | puris-AB097455 | 42 | asteroides-Z82227 | 36 | crassostreae-U92799 | 41 |
| jiangxiensis-AY639902 | 52 | arthritidis-AB212949 | 43 | arthritidis-AB108781 | 36 | crassostreae-Z37989 | 41 |
| nova-DQ840030 | 52 | asteroides-Z82228 | 44 | arthritidis-DQ659896 | 36 | seriolae-AF380936 | 42 |
| jiangxiensis-DQ840027 | 52 | beijingensis-AF154129 | 44 | mexicana-AY555577 | 37 | seriolae-X80592 | 43 |
| nova-DQ840028 | 52 | beijingensis-AB094640 | 44 | mexicana-AY560655 | 37 | seriolae-AY846841 | 43 |
| nova-DQ840029 | 52 | beijingensis-AB094646 | 44 | transvalensis-AB084447 | 38 | seriolae-AF254420 | 43 |
| farcinica-AJ131211 | 53 | beijingensis-DQ659901 | 44 | transvalensis-AB084448 | 38 | seriolae-AB255702 | 43 |
| farcinica-X91041 | 53 | beijingensis-AB094639 | 44 | asteroides-Z82220 | 38 | seriolae-AF254421 | 43 |
| farcinica-EF204470 | 53 | beijingensis-AB094645 | 44 | asteroides-Z82221 | 38 | seriolae-AF380937 | 43 |
| farcinica-AB162794 | 53 | beijingensis-AB094648 | 44 | asteroides-Z82229 | 38 | seriolae-EF192033 | 43 |
| shimofusensis-AB108777 | 54 | beijingensis-AB094656 | 44 | asteroides-AY191251 | 38 | seriolae-AF380938 | 43 |
| shimofusensis-AB108775 | 54 | beijingensis-AB094651 | 44 | asteroides-DQ659899 | 38 | seriolae-AY017474 | 43 |
| shimofusensis-AB108776 | 54 | beijingensis-AB094644 | 44 | transvalensis-AB084445 | 38 | seriolae-AF430039 | 43 |
| vermiculata-AB126873 | 55 | beijingensis-AB094654 | 44 | transvalensis-AB084446 | 38 | seriolae-Z36925 | 43 |
| vaccinii-X80597 | 55 | beijingensis-AB162629 | 44 | transvalensis-AB084444 | 38 | seriolae-DQ659915 | 43 |
| vaccinii-AF430045 | 55 | beijingensis-AB094653 | 44 | wallacei-EU099357 | 38 | seriolae-AB255699 | 43 |
| vaccinii-Z36927 | 55 | beijingensis-AB094643 | 44 | aobensis-AB126879 | 39 | seriolae-AB255700 | 43 |
| vaccinii-AY191252 | 55 | beijingensis-AB094642 | 44 | aobensis-AB126875 | 39 | seriolae-AB255701 | 43 |
| vaccinii-DQ659917 | 55 | beijingensis-AB094641 | 44 | aobensis-AB126876 | 39 | seriolae-AF251566 | 43 |
| lijiangensis-AY779043 | 56 | beijingensis-AB094649 | 44 | aobensis-AB126877 | 39 | seriolae-AF254418 | 43 |
| xishanensis-AY333115 | 56 | beijingensis-AB094655 | 44 | crassostreae-AF430049 | 40 | concava-EF177464 | 43 |
| gamkensis-DQ235272 | 56 | beijingensis-AB094652 | 44 | crassostreae-U92799 | 40 | concava-AB126880 | 43 |
| exalbida-AB187522 | 56 | araoensis-AB108779 | 44 | crassostreae-Z37989 | 40 | concava-AB126881 | 43 |
| exalbida-AB187521 | 56 | asteroides-Z82227 | 44 | alba-EU249584 | 41 | areane-DQ282122 | 44 |
| seriolae-AF380936 | 57 | arthritidis-AB108781 | 44 | ninae-DQ235687 | 41 | harenae-DQ282122 | 44 |
| nova-AB162788 | 58 | arthritidis-DQ659896 | 44 | amamiensis-AB275164 | 42 | transvalensis-AB084447 | 45 |
| otitidiscaviarum-M59056 | 59 | yamanashiensis-DQ659920 | 45 | vaccinii-X80597 | 43 | transvalensis-AB084448 | 45 |
| otitidiscaviarum-AB110907 | 59 | yamanashiensi-AB092561 | 46 | vaccinii-AF430045 | 43 | asteroides-Z82220 | 46 |
| otitidiscaviarum-X80599 | 59 | inohanensis-AJ619769 | 46 | vaccinii-Z36927 | 43 | asteroides-Z82221 | 46 |
| otitidiscaviarum-EU031786 | 59 | inohanensis-AB092560 | 46 | vaccinii-AY191252 | 43 | asteroides-Z82229 | 46 |
| otitidiscaviarum-AF430067 | 59 | inohanensis-DQ659908 | 46 | vaccinii-DQ659917 | 43 | asteroides-AY191251 | 46 |
| otitidiscaviarum-DQ659912 | 59 | otitidiscaviarum-M59056 | 47 | jinanensis-DQ462650 | 44 | asteroides-DQ659899 | 46 |
| otitidiscaviarum-AF430068 | 59 | otitidiscaviarum-AB110907 | 47 | speluncae-AM422449 | 44 | transvalensis-AB084445 | 46 |
| otitidiscaviarum-EU203569 | 59 | otitidiscaviarum-X80599 | 47 | elegans-DQ659905 | 45 | transvalensis-AB084446 | 46 |
| otitidiscaviarum-EU203570 | 59 | otitidiscaviarum-EU031786 | 47 | elegans-AB237142 | 45 | transvalensis-AB084444 | 46 |
| asiatica-AB162797 | 60 | otitidiscaviarum-AF430067 | 47 | otitidiscaviarum-Z82234 | 46 | wallacei-EU099357 | 46 |
| asiatica-AB097456 | 60 | otitidiscaviarum-DQ659912 | 47 | otitidiscaviarum-Z82238 | 46 | transvalensis-X80598 | 47 |
| asiatica-AB092570 | 60 | otitidiscaviarum-AF430068 | 47 | cyriacigeorgica-AB115948 | 47 | transvalensis-X80609 | 47 |
| asiatica-AB097457 | 60 | otitidiscaviarum-EU203569 | 47 | cyriacigeorgica-AJ508414 | 47 | transvalensis-AF430047 | 47 |
| asiatica-AB162798 | 60 | otitidiscaviarum-EU203570 | 47 | cyriacigeorgica-AB094577 | 47 | transvalensis-DQ659916 | 47 |
| asiatica-AB162796 | 60 | seriolae-AF380936 | 48 | cyriacigeorgica-AB094572 | 47 | transvalensis-Z36926 | 47 |
| asiatica-AB097458 | 60 | seriolae-X80592 | 48 | yamanashiensi-AB092561 | 48 | transvalensis-Z82232 | 47 |
| cyriacigeorgica-EF127500 | 61 | seriolae-AY846841 | 48 | inohanensis-AJ619769 | 48 | transvalensis-Z82240 | 48 |
| cyriacigeorgica-AB094578 | 61 | seriolae-AF254420 | 48 | inohanensis-AB092560 | 48 | transvalensis-Z82235 | 48 |
| cyriacigeorgica-AB115955 | 61 | seriolae-AB255702 | 48 | inohanensis-DQ659908 | 48 | transvalensis-Z82236 | 48 |
| cyriacigeorgica-EF127498 | 61 | seriolae-AF254421 | 48 | shimofusensis-AB108777 | 49 | blacklockiae-EU099360 | 49 |
| cyriacigeorgica-EF127499 | 61 | seriolae-AF380937 | 48 | shimofusensis-AB108775 | 49 | transvalensis-Z82233 | 49 |
| cyriacigeorgica-EF127501 | 61 | seriolae-EF192033 | 48 | shimofusensis-AB108776 | 49 | vermiculata-AB126873 | 50 |
| cyriacigeorgica-EF127502 | 61 | seriolae-AF380938 | 48 | elegans-AJ854057 | 50 | vaccinii-X80597 | 51 |
| transvalensis-AB201301 | 62 | seriolae-AY017474 | 48 | elegans-AJ854058 | 50 | vaccinii-AF430045 | 51 |
| transvalensis-AB201300 | 62 | seriolae-AF430039 | 48 | testacea-AB121769 | 51 | vaccinii-Z36927 | 51 |
| brasiliensis-AB201298 | 62 | seriolae-Z36925 | 48 | sienata-AB121770 | 51 | vaccinii-AY191252 | 51 |
| brasiliensis-AB201299 | 62 | seriolae-DQ659915 | 48 | testacea-AB192415 | 51 | vaccinii-DQ659917 | 51 |
| otitidiscaviarum-AB201303 | 62 | seriolae-AB255699 | 48 | flavorosea-AF430048 | 51 | cerradoensis-AF060790 | 52 |
| altamirensis-EU006090 | 63 | seriolae-AB255700 | 48 | flavorosea-Z46754 | 51 | aobensis-AB126878 | 52 |
| globerula-AF430065 | 64 | seriolae-AB255701 | 48 | asteroides-AF163818 | 51 | aobensis-AB126879 | 52 |
| globerula-DQ525592 | 64 | seriolae-AF251566 | 48 | asteroides-Z82230 | 51 | aobensis-AB126875 | 52 |
| corynebacterioides-AF430066 | 64 | seriolae-AF254418 | 48 | puris-AB097453 | 52 | aobensis-AB126876 | 52 |
| corynebacterioides-AY438619 | 64 | beijingensis-AB094650 | 49 | puris-AB097454 | 52 | aobensis-AB126877 | 52 |
| beijingensis-AB094650 | 65 | beijingensis-AB094647 | 49 | puris-AJ508748 | 52 | veterana-AF430059 | 52 |
| beijingensis-AB094647 | 65 | beijingensis-AB162628 | 49 | puris-AB097455 | 52 | kruczakiae-AY441974 | 52 |
| beijingensis-AB162628 | 65 | thailandica-AB126874 | 50 | carnea-X80602 | 53 | kruczakiae-DQ659909 | 52 |
| farcinica-X80595 | 66 | asteroides-AF430025 | 51 | carnea-AF430035 | 53 | veterana-AF278572 | 52 |
| farcinica-X80604 | 66 | asteroides-AF430026 | 51 | carnea-X80607 | 53 | veterana-AF430055 | 52 |
| farcinica-Z36936 | 66 | asteroides-X84850 | 51 | carnea-Z36929 | 53 | veterana-DQ659918 | 52 |
| farcinica-X80610 | 66 | asteroides-X80606 | 51 | carnea-AF430036 | 53 | veterana-AY191253 | 52 |
| farcinica-EF452728 | 66 | asteroides-Z36934 | 51 | carnea-AF430037 | 53 | veterana-AY171039 | 52 |
| farcinica-AF430034 | 66 | asteroides-AF430019 | 51 | yamanashiensis-DQ659920 | 54 | veterana-AF490540 | 52 |
| farcinica-AB162792 | 66 | nova-AB292584 | 51 | jiangxiensis-AY639902 | 55 | veterana-AY149599 | 52 |
| farcinica-AB162793 | 66 | asteroides-DQ659898 | 51 | nova-DQ840030 | 55 | elegans-AJ854057 | 53 |
| farcinica-AY640108 | 66 | neocaledoniensis-AY282603 | 52 | jiangxiensis-DQ840027 | 55 | elegans-DQ659905 | 53 |
| farcinica-AY640109 | 66 | coubleae-DQ235688 | 53 | nova-DQ840028 | 55 | elegans-AJ854058 | 53 |
| farcinica-AY640110 | 66 | pseudobrasiliensis-AB086862 | 54 | nova-DQ840029 | 55 | elegans-AB237142 | 53 |
| farcinica-AY640111 | 66 | pseudobrasiliensis-AB086861 | 55 | polyresistens-AY626158 | 56 | africana-AF277198 | 54 |
| farcinica-AY640112 | 66 | pseudobrasiliensis-AB080196 | 55 | cyriacigeorgica-AB094576 | 57 | africana-AF430054 | 54 |
| otitidiscaviarum-X80611 | 66 | pseudobrasiliensis-AF430043 | 55 | pseudobrasiliensis-AB086861 | 58 | africana-AF302232 | 54 |
| farcinica-AF430033 | 66 | pseudobrasiliensis-AF430042 | 55 | pseudobrasiliensis-AB080196 | 58 | africana-AY089701 | 54 |
| farcinica-AB162791 | 66 | pseudobrasiliensis-DQ659914 | 55 | pseudobrasiliensis-AF430043 | 58 | yamanashiensis-DQ659920 | 55 |
| farcinica-AB162795 | 66 | pseudobrasiliensis-X84853 | 55 | pseudobrasiliensis-AF430042 | 58 | yamanashiensi-AB092561 | 56 |
| farcinica-DQ659906 | 66 | pseudobrasiliensis-X84855 | 55 | pseudobrasiliensis-DQ659914 | 58 | inohanensis-AJ619769 | 56 |
| pseudobrasiliensis-AB086861 | 67 | pseudobrasiliensis-X84854 | 55 | pseudobrasiliensis-X84853 | 58 | inohanensis-AB092560 | 56 |
| pseudobrasiliensis-AB080196 | 67 | pseudobrasiliensis-X84852 | 55 | pseudobrasiliensis-X84855 | 58 | inohanensis-DQ659908 | 56 |
| pseudobrasiliensis-AF430043 | 67 | areane-DQ282122 | 56 | pseudobrasiliensis-X84854 | 58 | uniformis-AF430044 | 57 |
| pseudobrasiliensis-AF430042 | 67 | harenae-DQ282122 | 56 | pseudobrasiliensis-X84852 | 58 | uniformis-Z46752 | 57 |
| pseudobrasiliensis-DQ659914 | 67 | corynebacterioides-AF430066 | 57 | transvalensis-AB201302 | 59 | niigatensis-AB092562 | 58 |
| pseudobrasiliensis-X84853 | 67 | corynebacterioides-AY438619 | 57 | otitidiscaviarum-AB201303 | 59 | niigatensis-AB092563 | 58 |
| pseudobrasiliensis-X84855 | 67 | globerula-AF430065 | 58 | brasiliensis-X80591 | 60 | niigatensis-AB092564 | 58 |
| pseudobrasiliensis-X84854 | 67 | globerula-DQ525592 | 58 | brasiliensis-AY245543 | 60 | niigatensis-AB092565 | 58 |
| pseudobrasiliensis-X84852 | 67 | shimofusensis-AB108777 | 59 | brasiliensis-Z36935 | 60 | niigatensis-DQ659910 | 58 |
| transvalensis-AB084447 | 68 | shimofusensis-AB108775 | 59 | brasiliensis-AF430038 | 60 | transvalensis-AB201301 | 59 |
| transvalensis-AB084448 | 68 | shimofusensis-AB108776 | 59 | brasiliensis-X80608 | 60 | transvalensis-AB201302 | 60 |
| asteroides-Z82220 | 68 | alba-EU249584 | 60 | brasiliensis-DQ659902 | 60 | transvalensis-AB201300 | 60 |
| asteroides-Z82221 | 68 | ninae-DQ235687 | 60 | asteroides-Z82231 | 61 | brasiliensis-AB201298 | 60 |
| asteroides-Z82229 | 68 | transvalensis-AB084447 | 61 | transvalensis-X80598 | 62 | brasiliensis-AB201299 | 60 |
| asteroides-AY191251 | 68 | transvalensis-AB084448 | 61 | transvalensis-X80609 | 62 | otitidiscaviarum-AB201303 | 60 |
| asteroides-DQ659899 | 68 | asteroides-Z82220 | 61 | transvalensis-AF430047 | 62 | otitidiscaviarum-M59056 | 61 |
| transvalensis-AB084445 | 68 | asteroides-Z82221 | 61 | transvalensis-DQ659916 | 62 | otitidiscaviarum-AB110907 | 61 |
| transvalensis-AB084446 | 68 | asteroides-Z82229 | 61 | transvalensis-Z36926 | 62 | otitidiscaviarum-X80599 | 61 |
| transvalensis-AB084444 | 68 | asteroides-AY191251 | 61 | transvalensis-Z82232 | 62 | otitidiscaviarum-EU031786 | 61 |
| wallacei-EU099357 | 68 | asteroides-DQ659899 | 61 | neocaledoniensis-AY282603 | 63 | otitidiscaviarum-AF430067 | 61 |
| otitidiscaviarum-Z82234 | 69 | transvalensis-AB084445 | 61 | alba-AY222321 | 64 | otitidiscaviarum-DQ659912 | 61 |
| otitidiscaviarum-Z82238 | 69 | transvalensis-AB084446 | 61 | areane-DQ282122 | 65 | otitidiscaviarum-AF430068 | 61 |
| cerradoensis-AF060790 | 69 | transvalensis-AB084444 | 61 | harenae-DQ282122 | 65 | otitidiscaviarum-EU203569 | 61 |
| asteroides-AF430025 | 70 | wallacei-EU099357 | 61 | anaemiae-AB162801 | 66 | otitidiscaviarum-EU203570 | 61 |
| asteroides-AF430026 | 70 | abscessus-AF218293 | 62 | vinacea-AB162802 | 66 | miyunensis-AY639901 | 62 |
| asteroides-X84850 | 70 | asteroides-X84851 | 62 | vinacea-AB024312 | 66 | jiangxiensis-AY639902 | 63 |
| asteroides-X80606 | 70 | asteroides-Z82219 | 62 | vinacea-DQ659919 | 66 | nova-DQ840030 | 63 |
| asteroides-Z36934 | 70 | abscessus-AB108774 | 62 | miyunensis-AY639901 | 67 | nova-AB162788 | 64 |
| asteroides-AF430019 | 70 | abscessus-AB162809 | 62 | acidivorans-AM402972 | 67 | nova-X80593 | 64 |
| nova-AB292584 | 70 | abscessus-AB108771 | 62 | takedensis-AB158277 | 68 | nova-AB162789 | 64 |
| asteroides-DQ659898 | 70 | abscessus-AF430018 | 62 | takedensis-AB158278 | 68 | nova-AB162785 | 64 |
| nova-AB162785 | 71 | abscessus-AF218292 | 62 | takedensis-DQ840025 | 68 | nova-AF430030 | 64 |
| nova-DQ840026 | 71 | abscessus-DQ351151 | 62 | gamkensis-DQ235272 | 69 | nova-DQ840026 | 64 |
| nova-AB162787 | 71 | abscessus-DQ659895 | 62 | exalbida-AB187522 | 69 | nova-AB162783 | 64 |
| abscessus-AB108774 | 72 | abscessus-AB115182 | 62 | exalbida-AB187521 | 69 | nova-AF430031 | 64 |
| abscessus-AB162805 | 72 | abscessus-AB108773 | 62 | caishijiensis-AF459443 | 70 | nova-AF430029 | 64 |
| soli-AF277223 | 73 | abscessus-AB162805 | 62 | asteroides-AF430025 | 71 | nova-AB162790 | 64 |
| brevicatena-X80600 | 74 | abscessus-AB162808 | 62 | asteroides-AF430026 | 71 | nova-AF430028 | 64 |
| brevicatena-AF430040 | 74 | abscessus-AB162806 | 62 | asteroides-X84850 | 71 | nova-Z36930 | 64 |
| brevicatena-DQ659903 | 74 | abscessus-AB108770 | 62 | asteroides-X80606 | 71 | nova-AB162787 | 64 |
| paucivorans-AJ437308 | 74 | abscessus-AB162807 | 62 | asteroides-Z36934 | 71 | nova-DQ659911 | 64 |
| paucivorans-AF430041 | 74 | abscessus-AB212947 | 62 | asteroides-AF430019 | 71 | nova-AY191250 | 64 |
| paucivorans-AF179865 | 74 | vermiculata-AB126873 | 63 | nova-AB292584 | 71 | nova-AB162786 | 64 |
| paucivorans-DQ659913 | 74 | vaccinii-X80597 | 63 | asteroides-DQ659898 | 71 | nova-AF430032 | 64 |
| transvalensis-X80598 | 75 | vaccinii-AF430045 | 63 | xishanensis-AY333115 | 72 | nova-AB162784 | 64 |
| transvalensis-X80609 | 75 | vaccinii-Z36927 | 63 | vermiculata-AB126873 | 73 | jiangxiensis-DQ840027 | 64 |
| transvalensis-AF430047 | 75 | vaccinii-AY191252 | 63 | tenerifensis-AJ556157 | 74 | nova-DQ840028 | 64 |
| transvalensis-DQ659916 | 75 | vaccinii-DQ659917 | 63 | brevicatena-X80600 | 75 | nova-DQ840029 | 64 |
| transvalensis-Z36926 | 75 | cerradoensis-AF060790 | 64 | brevicatena-AF430040 | 75 | anaemiae-AB162801 | 65 |
| transvalensis-Z82232 | 75 | nova-AB162788 | 65 | brevicatena-DQ659903 | 75 | pseudovaccinii-AF430046 | 66 |
| nova-AB162789 | 76 | pseudovaccinii-AF430046 | 66 | paucivorans-AJ437308 | 75 | vinacea-AB162802 | 66 |
| nova-AF430030 | 76 | takedensis-AB158277 | 67 | paucivorans-AF430041 | 75 | vinacea-AB024312 | 66 |
| nova-AF430031 | 76 | takedensis-AB158278 | 67 | paucivorans-AF179865 | 75 | vinacea-DQ659919 | 66 |
| nova-AF430029 | 76 | takedensis-DQ840025 | 67 | paucivorans-DQ659913 | 75 | acidivorans-AM402972 | 66 |
| nova-AF430028 | 76 | asiatica-AB092568 | 68 | transvalensis-Z82240 | 76 | pseudobrasiliensis-AB086861 | 67 |
| nova-Z36930 | 76 | cyriacigeorgica-AB094576 | 69 | transvalensis-Z82235 | 76 | pseudobrasiliensis-AB086862 | 67 |
| carnea-X80602 | 77 | jejuensis-AY964666 | 70 | transvalensis-Z82236 | 76 | pseudobrasiliensis-AB080196 | 67 |
| carnea-AF430035 | 77 | asteroides-X53205 | 71 | blacklockiae-EU099360 | 76 | pseudobrasiliensis-AF430043 | 67 |
| carnea-X80607 | 77 | seriolae-AB060281 | 72 | transvalensis-Z82233 | 76 | pseudobrasiliensis-AF430042 | 67 |
| carnea-Z36929 | 77 | seriolae-AB060282 | 73 | lijiangensis-AY779043 | 77 | pseudobrasiliensis-DQ659914 | 67 |
| carnea-AF430036 | 77 | pneumoniae-AB108780 | 74 | brevicatena-Z36928 | 78 | pseudobrasiliensis-X84853 | 67 |
| carnea-AF430037 | 77 | soli-AF277223 | 75 | thailandica-AB126874 | 79 | pseudobrasiliensis-X84855 | 67 |
| flavorosea-AF430048 | 77 | pigrifrangens-AF219974 | 76 | altamirensis-EU006090 | 80 | pseudobrasiliensis-X84854 | 67 |
| flavorosea-Z46754 | 77 | seriolae-EF513204 | 77 | iowensis-DQ925490 | 80 | pseudobrasiliensis-X84852 | 67 |
