# Supplementary material for: Defining Reference Sequences for Nocardia Species by Similarity and Clustering Analyses of 16S rRNA Gene Sequence Data
Source: PLoS One. 2011 Jun 8;6(6):e19517. doi: 10.1371/journal.pone.0019517 (PMC3110597; doi:10.1371/journal.pone.0019517)
Supplement: Table S4 — Classification of Nocardia species using different machine learning methods. (DOC) [file pone.0019517.s006.doc]

**Table S4. Classification of *Nocardia* species using different machine learning methods**

| **Strain** | **Simple KNN** | **Matlab NBayes** | **Matlab KNN** | **Alignment** | **Weka NBayes** | **Weka KNN** | **Consensus** |
| --- | --- | --- | --- | --- | --- | --- | --- |
| AB035565 | seriolae_4 | seriolae_4 | seriolae_4 | seriolae_2 | seriolae_4 | seriolae_4 | seriolae |
| AB092437 | jejuensis/ alba | jejuensis/ alba | concava_2 | inohanensis | seriolae_4 | brasiliensis | jejuensis/ alba |
| AB092438 | elegans/ africana | elegans/ africana | elegans/ africana | elegans/ africana | veterana_2 | elegans/ africana | elegans/ africana |
| AB126882 | concava | concava | concava_2 | concava_2 | seriolae_4 | concava_2 | concava |
| AB126883 | concava_2 | concava_2 | concava_2 | concava_2 | seriolae_4 | cerradoensis | concava |
| AB187521 | exalbida | exalbida | exalbida | exalbida | exalbida | elegans/ africana | exalbida |
| AB187522 | exalbida | exalbida | exalbida | exalbida | exalbida | exalbida | exalbida |
| AB242562 | mexicana | mexicana | transvalensis/ brasiliensis | mexicana | mexicana | polyresistens/ lijiangensis/ xishanensis | mexicana |
| AB243007 | globerula | corynebacterioides | globerula | mexicana | seriolae | globerula | globerula |
| AB284122 | beijingensis | pneumoniae | beijingensis | beijingensis | asteroides_5 | beijingensis_2 | beijingensis |
| AF227864 | globerula | corynebacterioides | corynebacterioides | corynebacterioides | seriolae | globerula | corynebacterioides |
| AF277203 | salmonicida | salmonicida | salmonicida | fluminea | soli | salmonicida | salmonicida |
| AF277205 | fluminea | fluminea | soli | fluminea_2 | soli/ coubleae | fluminea | fluminea |
| AF277206 | fluminea | fluminea | soli | fluminea_2 | crassostreae | fluminea | fluminea |
| AF277207 | fluminea | salmonicida | salmonicida | fluminea | soli | flavorosea | salmonicida/ fluminea |
| AF277208 | fluminea | salmonicida | soli | fluminea | soli | flavorosea | fluminea/ soli |
| AF277209 | fluminea | salmonicida | soli | fluminea | soli | flavorosea | fluminea/ soli |
| AF277210 | soli | soli | soli | soli | soli/ coubleae | soli/ coubleae | soli |
| AF277211 | soli | soli | soli | soli | flavorosea | soli/ coubleae | soli |
| AF277212 | salmonicida | salmonicida | soli | salmonicida | flavorosea | puris | salmonicida |
| AF277213 | soli | soli | soli | soli | flavorosea | soli/ coubleae | soli |
| AF277214 | soli | soli | soli | soli | soli/ coubleae | crassostreae | soli |
| AF277215 | soli | soli | soli | soli | soli/ coubleae | soli/ coubleae | soli |
| AF277216 | soli | soli | soli | soli | ignorata | soli/ coubleae | soli |
| AF277217 | soli | soli | soli | soli | soli/ coubleae | crassostreae | soli |
| AF277218 | soli/ coubleae | soli/ coubleae | salmonicida | fluminea | soli | corynebacterioides | soli |
| AF277219 | salmonicida | salmonicida | soli | soli | flavorosea | puris | salmonicida/ soli |
| AF277220 | soli | soli | soli | soli | flavorosea | soli/ coubleae | soli |
| AF277221 | soli | soli | soli | soli | soli/ coubleae | crassostreae | soli |
| AF277222 | salmonicida | salmonicida | salmonicida | soli | soli | salmonicida | salmonicida |
| AF277224 | fluminea | salmonicida | salmonicida | fluminea | soli | flavorosea | salmonicida/ fluminea |
| AF277225 | soli | soli | soli | soli | soli/ coubleae | soli/ coubleae | soli |
| AF277226 | soli | soli | soli | soli | soli/ coubleae | soli/ coubleae | soli |
| AF277227 | soli | soli | soli | fluminea_2 | soli/ coubleae | soli/ coubleae | soli |
| AF331799 | asteroides_3 | asteroides_3 | asteroides_4 | asteroides_2 | asteroides_3 | asteroides_3 | asteroides |
| AF397063 | niigatensis | niigatensis | niigatensis | niigatensis | niigatensis | niigatensis | niigatensis |
| AF421563 | seriolae_4 | seriolae_4 | seriolae_4 | seriolae_2 | seriolae_4 | seriolae_4 | seriolae |
| AF421564 | vinacea | transvalensis_3 | transvalensis/ brasiliensis | otitidiscaviarum_2 | seriolae_4 | pseudobrasiliensis_2 | transvalensis |
| AF421565 | pseudobrasiliensis | pseudobrasiliensis_2 | pseudobrasiliensis_2 | pseudobrasiliensis | pseudobrasiliensis_2 | pseudobrasiliensis_2 | pseudobrasiliensis |
| AF430021 | flavorosea | flavorosea | carnea | carnea | carnea | farcinica | carnea |
| AF430022 | flavorosea | flavorosea | carnea | carnea | carnea | farcinica | carnea |
| AF430023 | flavorosea | flavorosea | carnea | carnea | carnea | farcinica | carnea |
| AF430024 | asteroides/ jinanensis/ speluncae | carnea | carnea | asteroides/ jinanensis/ speluncae | testacea | asteroides_2 | asteroides |
| AF430056 | tenerifensis/ altamirensis | brasiliensis | brasiliensis | areane/ harenae | farcinica | jejuensis/ alba | brasiliensis |
| AF430057 | polyresistens/ lijiangensis/ xishanensis | asteroides_4 | asteroides_4 | iowensis_2 | asteroides_4 | asteroides_5 | asteroides |
| AF430058 | concava_2 | concava_2 | concava_2 | concava_2 | seriolae_4 | concava_2 | concava |
| AF430060 | niigatensis | niigatensis | niigatensis | niigatensis_2 | niigatensis | niigatensis | niigatensis |
| AF430061 | acidivorans | acidivorans | acidivorans | pseudobrasiliensis_2 | polyresistens/ lijiangensis/ xishanensis | abscessus | acidivorans |
| AF430062 | puris | puris | puris | puris | arthritidis | puris | puris |
| AF430063 | soli | soli | soli | soli | soli/ coubleae | soli/ coubleae | soli |
| AF430064 | mexicana | mexicana | mexicana | mexicana | polyresistens/ lijiangensis/ xishanensis | mexicana | mexicana |
| AF487704 | globerula | globerula | globerula | globerula | globerula | gamkensis | globerula |
| AJ786787 | cyriacigeorgica_2 | cyriacigeorgica_2 | cyriacigeorgica_3 | cyriacigeorgica_3 | cyriacigeorgica_3 | asteroides_4 | cyriacigeorgica |
| AJ971864 | seriolae_2 | seriolae_2 | globerula | corynebacterioides | seriolae | seriolae_4 | seriolae |
| AM411940 | nova | nova | nova | nova | nova | nova | nova |
| AM411941 | nova | nova | nova | nova | nova | miyunensis/ jiangxiensis/ nova | nova |
| AM411942 | nova | nova | nova | nova | nova | nova | nova |
| AM411943 | nova | nova | nova | nova | nova | miyunensis/ jiangxiensis/ nova | nova |
| AM411944 | abscessus | abscessus | abscessus | abscessus_2 | abscessus | cyriacigeorgica_2 | abscessus |
| AM411945 | abscessus | abscessus | abscessus | abscessus_2 | abscessus | cyriacigeorgica_2 | abscessus |
| AM411946 | abscessus | abscessus | abscessus | abscessus_2 | abscessus | cyriacigeorgica_2 | abscessus |
| AM411947 | abscessus | abscessus | abscessus | abscessus_2 | abscessus | cyriacigeorgica_2 | abscessus |
| AM411948 | abscessus | abscessus | abscessus | abscessus_2 | abscessus | cyriacigeorgica_2 | abscessus |
| AM411949 | thailandica/ neocaledoniensis | thailandica/ neocaledoniensis | thailandica/ neocaledoniensis | abscessus | asteroides_5 | miyunensis/ jiangxiensis/ nova | thailandica/ neocaledoniensis |
| AM411950 | abscessus | abscessus | abscessus | abscessus | abscessus | abscessus | abscessus |
| AM411951 | arthritidis | arthritidis | arthritidis | beijingensis_2 | arthritidis | aobensis | arthritidis |
| AM411952 | arthritidis | arthritidis | arthritidis | beijingensis_2 | arthritidis | areane/ harenae | arthritidis |
| AM411953 | arthritidis | arthritidis | arthritidis | beijingensis_2 | arthritidis | aobensis | arthritidis |
| AM411954 | arthritidis | arthritidis | arthritidis | beijingensis_2 | arthritidis | areane/ harenae | arthritidis |
| AM489699 | beijingensis | puris | puris | alba_2 | arthritidis | beijingensis_2 | puris/ beijingensis |
| AY155203 | asteroides_5 | asteroides_5 | asteroides_5 | transvalensis/ asteroides | vinacea | asteroides_5 | asteroides |
| AY524857 | farcinica | farcinica | farcinica | farcinica | farcinica_2 | farcinica | farcinica |
| AY524858 | farcinica | farcinica | farcinica | farcinica | farcinica_2 | farcinica | farcinica |
| AY524859 | farcinica | farcinica_2 | farcinica | farcinica | farcinica | farcinica_2 | farcinica |
| AY524860 | farcinica | farcinica_2 | farcinica | farcinica | farcinica_2 | farcinica_2 | farcinica |
| AY524861 | farcinica | farcinica | farcinica | farcinica | farcinica_2 | farcinica | farcinica |
| AY996839 | salmonicida | salmonicida | soli | salmonicida | flavorosea | puris | salmonicida |
| DQ008603 | abscessus | abscessus | abscessus | abscessus_2 | abscessus | abscessus | abscessus |
| DQ448718 | arthritidis | arthritidis | arthritidis | abscessus_2 | beijingensis_2 | aobensis | arthritidis |
| DQ925490 | brasiliensis | tenerifensis/ altamirensis | brasiliensis | iowensis | brasiliensis | inohanensis | brasiliensis |
| EF212021 | fluminea | fluminea | soli | fluminea_2 | testacea | fluminea | fluminea |
| EF212023 | ninae/ alba | ninae/ alba | ninae/ alba | alba | testacea | elegans/ africana | alba |
| EF216351 | soli | fluminea | soli | fluminea_2 | flavorosea | crassostreae | fluminea/ soli |
| EF216365 | asteroides/ jinanensis/ speluncae | asteroides/ jinanensis/ speluncae | asteroides/ jinanensis/ speluncae | asteroides/ jinanensis/ speluncae | testacea | soli | asteroides/ jinanensis/ speluncae |
| EF216366 | fluminea | fluminea | soli | fluminea_2 | flavorosea | fluminea | fluminea |
| EF216367 | salmonicida | salmonicida | soli | salmonicida | flavorosea | puris | salmonicida |
| EF502100 | carnea | carnea | carnea | carnea | testacea | mexicana | carnea |
| EF538720 | higoensis | higoensis | farcinica | shimofusensis | farcinica | areane/ harenae | farcinica/ higoensis |
| EF538722 | asteroides_4 | asteroides_4 | asteroides_4 | asteroides_3 | cyriacigeorgica_2 | asteroides_4 | asteroides |
| EF538728 | aobensis | aobensis | aobensis | aobensis | aobensis | aobensis | aobensis |
| EF538729 | elegans/ africana | elegans/ africana | aobensis | elegans/ africana | elegans/ africana | elegans/ africana | elegans/ africana |
| EF538730 | aobensis | aobensis | aobensis | aobensis | aobensis | aobensis | aobensis |
| EF538731 | carnea | asteroides_2 | carnea | carnea | carnea | carnea | carnea |
| EF538732 | nova | nova | nova | nova | nova | nova | nova |
| EF538735 | aobensis | aobensis | aobensis | aobensis | aobensis | aobensis | aobensis |
| EF538736 | elegans/ africana | elegans/ africana | elegans/ africana | elegans/ africana | elegans/ africana | cyriacigeorgica_3 | elegans/ africana |
| EF538737 | asteroides_2 | asteroides_2 | asteroides_2 | carnea | testacea | cyriacigeorgica_3 | asteroides |
| EF538738 | carnea | carnea | carnea | carnea | carnea | carnea | carnea |
| EF538741 | cerradoensis | cerradoensis | cerradoensis | transvalensis_4 | asteroides_5 | carnea | cerradoensis |
| EF546420 | niigatensis | niigatensis | niigatensis | niigatensis_2 | seriolae_4 | thailandica/ neocaledoniensis | niigatensis |
| EF546424 | anaemiae | anaemiae | vinacea | anaemiae | vinacea | amamiensis | anaemiae |
| EF546427 | niigatensis | niigatensis | niigatensis | niigatensis_2 | niigatensis | thailandica/ neocaledoniensis | niigatensis |
| EU119249 | thailandica/ neocaledoniensis | thailandica/ neocaledoniensis | thailandica/ neocaledoniensis | abscessus_2 | soli | testacea | thailandica/ neocaledoniensis |
| EU119250 | abscessus | abscessus | abscessus | abscessus | abscessus | abscessus | abscessus |
| EU119251 | carnea | carnea | carnea | carnea_2 | carnea | carnea | carnea |
| EU119252 | carnea | carnea | carnea | carnea_2 | carnea | carnea | carnea |
| EU119253 | thailandica/ neocaledoniensis | thailandica/ neocaledoniensis | asteroides/ jinanensis/ speluncae | asteroides_2 | soli | miyunensis/ jiangxiensis/ nova | asteroides |
| EU158360 | abscessus | thailandica/ neocaledoniensis | abscessus | abscessus | asteroides_5 | abscessus | abscessus |
| EU158361 | takedensis | takedensis | takedensis | takedensis | takedensis | takedensis | takedensis |
| EU158362 | testacea | asteroides/ jinanensis/ speluncae | testacea | carnea_2 | paucivorans | tenerifensis/ altamirensis | testacea |
